# miR-19a promotes colorectal cancer proliferation and migration by targeting TIA1

**DOI:** 10.1186/s12943-017-0625-8

**Published:** 2017-03-04

**Authors:** Yanqing Liu, Rui Liu, Fei Yang, Rongjie Cheng, Xiaorui Chen, Shufang Cui, Yuanyuan Gu, Wu Sun, Chaoying You, Zhijian Liu, Feng Sun, Yanbo Wang, Zheng Fu, Chao Ye, Chenyu Zhang, Jing Li, Xi Chen

**Affiliations:** 1 State Key Laboratory of Pharmaceutical Biotechnology, Collaborative Innovation Center of Chemistry for Life Sciences, Jiangsu Engineering Research Center for MicroRNA Biology and Biotechnology, NJU Advanced Institute for Life Sciences (NAILS), School of Life Sciences, Nanjing University, 163 Xianlin Road, Nanjing, Jiangsu 210046 China; 2 Tianjin Medical University Cancer Institute and Hospital, National Clinical Research Center of Cancer, Key Laboratory of Cancer Prevention and Therapy, Tianjin, 300060 China; 3 Department of Gastrointestinal Surgery, Nanjing Drum Tower Hospital Clinical College of Nanjing Medical University, 321 Zhongshan Road, Nanjing, Jiangsu 210008 China

**Keywords:** Colorectal cancer, microRNA, miR-19a, TIA1

## Abstract

**Background:**

Colorectal cancer (CRC) is a major worldwide health problem due to its high prevalence and mortality rate. T-cell intracellular antigen 1 (TIA1) is an important tumor suppressor involved in many aspects of carcinogenesis and cancer development. How TIA1 expression is regulated during CRC development remains to be carefully elucidated.

**Methods:**

In CRC tissue sample pairs, TIA1 protein and mRNA levels were monitored by Western blot and qRT-PCR, respectively. Combining meta-analysis and miRNA target prediction software, we could predict microRNAs that targeted TIA1. Next, three CRC cell lines (SW480, Caco2 and HT29) were used to demonstrate the direct targeting of TIA1 by miR-19a. In addition, we investigated the biological effects of TIA1 inhibition by miR-19a both in vitro by CCK-8, EdU, Transwell, Ki67 immunofluorescence and Colony formation assays and in vivo by a xenograft mice model.

**Results:**

In colorectal cancer (CRC), we found that TIA1 protein, but not its mRNA, was downregulated. We predicted that TIA1 was a target of miR-19a and validated that miR-19a binded directly to the 3’-UTR of TIA1 mRNA. miR-19a could promote cell proliferation and migration in CRC cells and accelerated tumor growth in xenograft mice by targeting TIA1.

**Conclusions:**

This study highlights an oncomiR role for miR-19a in regulating TIA1 in CRC and suggests that miR-19a may be a novel molecular therapeutic target for CRC.

**Electronic supplementary material:**

The online version of this article (doi:10.1186/s12943-017-0625-8) contains supplementary material, which is available to authorized users.

## Background

Colorectal cancer (CRC) is one of the most prevalent malignant tumors, with high morbidity and mortality worldwide. In the USA, CRC is currently the third most common cancer type and the third leading cause of cancer-related death [1]. Although advances in screening and treatment have improved the life expectancy of CRC patients in recent decades [2], CRC remains a major health problem all over the world. Much more attention should be given to the exact mechanisms contributing to the initiation and development of CRC.

Although there are many risk factors for CRC (including obesity, smoking, dietary patterns, physical inactivity, and genetic and epigenetic factors) [3–5], understanding the molecular basis of individual susceptibility to colorectal cancer and determining the factors that initiate the development of the tumor, drive its progression and determine its responsiveness or resistance to antitumor agents are the most important tasks in the study of this disease [2]. Among the myriad CRC-related molecular factors, oncogene activation (e.g., KRAS and IGF1R) and tumor suppressor gene silencing (e.g., APC and PDCD4) play vital roles during CRC tumorigenesis [6–9]. T-cell intracellular antigen 1 (TIA1) is an RNA binding protein and is linked to multiple biologic processes associated with RNA metabolism, both in the nucleus and in the cytoplasm [10]. TIA1 is thought to be a new member of the tumor suppressor family [11], as TIA1 regulates, modulates and/or interacts with many types of mRNA involved in cancer cell proliferation, apoptosis, angiogenesis, invasiveness and metastasis as well as in immune evasion [12–16]. For example, it has been reported that knockdown of TIA1 triggers cell proliferation and invasion as well as tumor growth [14]. Furthermore, TIA1 has been found to regulate many oncogenes (e.g., RAB40B) to inhibit cell proliferation [12]. Moreover, TIA1 can promote cell apoptosis by regulating Fas alternative splicing [17]. In CRC, TIA1 is also closely connected to tumorigenesis. For example, TIA1 has been found to regulate VEGF isoform expression, angiogenesis, tumor growth and bevacizumab resistance in CRC [15]. Moreover, TIA1 can be used to supplement prognostic information related to TNM stage and adjuvant therapy in mismatch repair-proficient colorectal cancer patients [16]. Because of the myriad of tumor suppressor functions of TIA1, it is imperative that we elucidate the mechanisms underlying how TIA1 is regulated during tumorigenesis, especially in CRC.

MicroRNAs (miRNAs) are small (19–23 nucleotides) non-coding RNA molecules [18] that act as endogenous suppressors of gene expression by binding to the 3’-untranslated region (3’-UTR) of target mRNAs to induce translational repression or mRNA cleavage. Occasionally, miRNAs may bind directly to coding sequence of mRNAs or even function as activators of gene expression by binding to the 5’-UTR of target mRNAs [19–21]. As vital post-transcriptional regulators, miRNAs are involved in numerous physiological and pathological processes, such as developmental timing [22], hematopoietic cell differentiation [23], cell proliferation [24], organ development [25] and tumorigenesis in particular [26, 27]. Many miRNAs are directly or indirectly correlated with cancer genes and can function as either tumor suppressor miRNAs or oncomiRs [27]. During CRC initiation and progression, some miRNAs show a significant alteration in their expression patterns and influence CRC cell proliferation, invasion and apoptosis. Among these miRNAs, miR-19a is one of the most important. miR-19a belongs to a well-known and important miRNA family named mir-17-92 (also known as oncomir-1) and is a miRNA polycistron with pleiotropic functions in cell survival, proliferation, differentiation and angiogenesis [28–31]. miR-19a has been reported to be significantly overexpressed in CRC [32]. Moreover, miR-19a has been found to be induced by PRL-3 to promote the proliferation and metastasis of CRC cells [33]. miR-19a can also enhance the invasion and metastasis of CRC cells by targeting TG2 [34]. More importantly, miR-19a is associated with lymph metastasis and mediates TNF-α-induced EMT in CRC [32]. These studies have revealed an important oncogenic role of miR-19a in CRC. However, the precise molecular mechanism through which miR-19a influences CRC progression remains unknown.

In this study, we identified TIA1 as a direct target gene of miR-19a in CRC. We also detected an inverse correlation between miR-19a and TIA1 protein levels in CRC tissues. Moreover, we provided evidence that miR-19a can promote CRC cell proliferation and migration in vitro and accelerate tumor growth in vivo by targeting TIA1.

## Methods

### Tissue samples

CRC tissue and paired normal adjacent tissue samples were acquired from patients undergoing a surgical procedure at the Affiliated Drum Tower Hospital of Nanjing University Medical School (Nanjing, China). Both the tumor and normal tissues were sent for histological analysis and diagnostic confirmation. Written consent was obtained from all patients, and all protocols concerning the use of patient samples in this study were approved by the Ethics Committee of Nanjing University. Tissue samples were immediately frozen in liquid nitrogen at the time of surgery and stored at -80 °C. All experiments were performed in accordance with The Code of Ethics of the World Medical Association (Declaration of Helsinki) and approved guidelines of the Nanjing University. The clinical features of the patients are listed in Additional file 1: Table S1.

### Cell culture

The three human CRC cell lines SW480, Caco2 and HT29 were purchased from the Shanghai Institute of Biochemistry and Cell Biology, Chinese Academy of Sciences (Shanghai, China). SW480 and HT29 cells were cultured in RPMI-1640 (Gibco, Carlsbad, CA, USA) supplemented with 10% fetal bovine serum (FBS, Gibco) in a humidified incubator at 37 °C with 5% CO_2_. Caco2 cells were cultured in DMEM (Gibco) supplemented with 10% FBS (Gibco) in a humidified incubator at 37 °C with 5% CO_2_.

### Protein isolation and western blot

RIPA lysis buffer (Beyotime, Shanghai, China) with freshly added PMSF (Beyotime, Shanghai, China) was used to isolate protein from cells or tissues. Proteins were separated by 10% SDS-PAGE (Bio-Rad). Antibodies against TIA1, CMYC, PDCD4 and GAPDH were purchased from Santa Cruz Biotechnology (sc-365349, sc-40, sc-130545 and sc-25778, respectively; Santa Cruz, CA, USA).

### Meta-analysis of miRNA expression in CRC

We used an online database YM500 to analyze and compare the miRNA expression in colon cancer tissues and normal solid tissues. This database compared the miRNA expression profiles between 8 normal solid colon tissues and 429 primary solid colon tumors, and a total of 2578 miRNAs were analyzed. In total, 273 miRNAs were found to be significantly changed in the solid tumors compared to the normal tissues (128 were upregulated, and 145 were downregulated, Additional file 2: Table S2). Among the 128 upregulated miRNAs, 26 miRNAs were determined to be upregulated by an infinite fold change at an extremely low expression level in the normal group of these miRNAs. For these miRNAs, we set a threshold of 26.18 for the base mean of the primary solid tumors, which was the average value of the base means of these miRNAs. Above this threshold, we found 7 significantly upregulated miRNAs, and miR-19a ranked first among them. For the remainder of the 102 upregulated miRNAs, we set a threshold of 42.46 for the fold change, which was the average fold change value of these miRNAs. Above this threshold, we found another 22 significantly upregulated miRNAs, and miR-19b ranked first among these. In sum, we obtained 29 significantly upregulated miRNAs, and miR-19a/b was at the top of the list (Additional file 3: Table S3).

### RNA isolation and quantitative RT-PCR

Total RNA was extracted from the cultured cells and tissues using Trizol Reagent (Invitrogen, CA, USA). To quantify mature miR-19a and 19b, TaqMan miRNA Assay Probes (Applied Biosystems, Foster City, CA) were used according to the manufacturer’s instructions. Quantitative real-time PCR was performed using a TaqMan PCR kit on an Applied Biosystems 7500 Sequence Detection System (Applied Biosystems). All of the reactions were run in triplicate. After the reactions were complete, the cycle threshold (C_T_) data were determined using fixed threshold settings, and the mean C_T_ was determined from triplicate PCRs. A comparative C_T_ method was used to compare each condition to the control reactions. U6 snRNA was used as an internal control, and the relative amount of miRNA normalized to U6 was calculated with the equation 2^-ΔΔCT^ in which ΔΔC_T_ = (C_T__miR-19a_ − C_T__U6_)_tumor_ − (C_T__miR-19a_ − C_T__U6_)_control_.

To quantify TIA1 and GAPDH mRNA, oligo d(T)18 primers (TaKaRa) were used to reverse transcribe total RNA into cDNA. Then, a qRT-PCR was run by using SYBR Green dye (Invitrogen) and specific primers for TIA1 and GAPDH. The primer sequences were as follows: TIA1 (sense): TCCCGCTCCAAAGAGTACATATGAG; TIA1 (antisense): AAACAATTGCATGTGCTGCACTTTC; GAPDH (sense): CGAGCCACATCGCTCAGACA; and GAPDH (antisense): GTGGTGAAGACGCCAGTGGA. After the reactions were complete, the C_T_ values were determined by setting a fixed threshold. The relative amounts of TIA1 mRNAs were normalized to GAPDH using a method similar to that described above.

### miRNA overexpression and knockdown

miRNA overexpression and knockdown was achieved by transfecting CRC cells with miRNA mimics or inhibitors, respectively. Synthetic miRNA mimics and inhibitors and scrambled negative control RNAs (control mimic and inhibitor) were purchased from GenePharma (Shanghai, China). SW480, Caco2 and HT29 cells were seeded in 6-well plates and transfected using Lipofectamine 2000 (Invitrogen) on the following day when the cells were approximately 60–80% confluent. For miRNA overexpression and knockdown, 200 pmol of miRNA mimic or inhibitor and corresponding negative control were added to each well. At 6 h after transfection, the SW480 and HT29 cell medium was changed to RPMI-1640 supplemented with 2% FBS, and the Caco2 cell medium was changed to DMEM supplemented with 2% FBS. The cells were harvested 48 h after transfection for total RNA or protein isolation.

### Plasmid construction and siRNA interference assay

Mammalian expression plasmids were purchased from Genescript (Nanjing, China). An empty plasmid served as a negative control (control plasmid). siRNAs designed to specifically silence TIA1 or c-MYC were purchased from GenePharma (Shanghai, China). A scrambled siRNA served as a control. The siRNA sequences were as follows: si-TIA1: TGCACAACAAATTGGCCAGTA and si-c-MYC: ACGGAACTCTTGTGCGTAA. The overexpression plasmids and siRNAs were transfected into CRC cells using Lipofectamine 2000 (Invitrogen). Total RNA and protein were isolated 48 h after transfection and were assessed by quantitative RT-PCR and western blot, respectively.

### Pull-down assay

For miRNA pull-down, SW480 cells which were transfected with biotinylated miR-19a (miR-19a probe) or control probe (Genescript, Nanjing, China) were harvested in lysis buffer (20 mM Tris pH 7.5, 100 mM KCl, 5 mM MgCl2, 0.5% NP-40 and 1 U/ul Recombinant RNAse inhibitor (TaKaRa)), and the total RNA was pretreated with DNaseI and then heated at 65 °C for 5 min, followed by an instant ice bath. Then the RNA was incubated with streptavidin-coated magnetic beads (New England BioLabs, S1420S) at 4 °C for 4 h, with constant rotation. After incubation, two washes with lysis buffer were performed and RNA was extracted with Trizol (Invitrogen, CA, USA).

For mRNA pull-down, two DNA probes complementary to TIA1 mRNA was synthesized with 3’ terminal biotin labels. A scrambled biotinylated probe was used as negative control (Genescript, Nanjing, China). The probes (8 pmol/ul) were then incubated with streptavidin-coated magnetic beads (New England BioLabs, S1420S) at 25 °C for 1 h to generate probe-coated magnetic beads. SW480 cells were harvested in lysis buffer as described above. Then the lysate was incubated with probe-coated beads at 37 °C for 3 h, with constant rotation. After incubation, two washes with lysis buffer were performed and RNA was extracted with Trizol (Invitrogen, CA, USA). The sequence of TIA1 probes were as follows: Probe 1: AAAATCTAAATTTTGCATATT; Probe 2: ATAAGAGAACAATGAGGGTCC.

The extracted RNAs were analyzed by qRT-PCR.

### Luciferase reporter assay

An 800-bp fragment of the TIA1 3’-UTR containing the two conserved miR-19a binding sites was inserted into a luciferase reporter plasmid (Genescript, Nanjing, China). To test binding specificity, sequences that interacted with the miR-19a seed sequence were mutated from TTGCACA to AACGTGT, and the synthetic TIA1 3’-UTR mutant fragment was inserted into an equivalent reporter plasmid (Genescript, Nanjing, China). For the luciferase reporter assays, SW480 cells were cultured in 24-well plates, and each well was co-transfected with 0.2 μg of firefly luciferase reporter plasmid, 0.2 μg of β-galactosidase (β-gal) expression plasmid (Ambion), and equal amounts (50 pmol) of miR-19a mimic, miR-19a inhibitor or the scrambled negative control RNAs using Lipofectamine 2000 (Invitrogen). The β-gal plasmid was used as a transfection efficiency control. The cells were assayed using a luciferase assay kit 24 h post-transfection (Promega, Madison, WI, USA).

### Cell proliferation assay

For CCK-8 assay, SW480 cells were plated at 2 × 10^4^ cells per well in 96-well plates and incubated overnight in RPMI-1640 supplemented with 10% FBS. The cell proliferation index was measured using a Cell Counting Kit-8 (CK04-500, Dojindo, Japan) at 12, 24, 36, 48, and 60 h post-transfection according to the manufacturer’s instruction. Absorbance was measured at a wavelength of 450 nm.

For EdU assay, SW480 cells were seeded in 48-well plates (Corning). After transfection, when the confluency of SW480 cells reached 80%, an EdU assay kit (RiBoBio, Guangzhou, China) was used to determine the proliferation rate of the cells. The manufacturer’s instruction was followed except that the nucleus staining dye was changed from Hoechst 33342 (supplied with the kit) to DAPI (Beyotime). After staining, the cells were captured by photomicroscopy (BX51 Olympus, Japan).

### Cell migration assay

Cell migration assays were performed using Millipore 24-well Millicell (Millipore) plates containing an 8-μm pore membrane. The bottom face of the membrane was coated with 10 μg/mL fibronectin (Gibco). Cells were harvested 24 h after transfection and suspended in FBS-free RPMI-1640 culture medium. The cells were then added to the upper chamber (2 × 10^4^ cells/well), and 0.5 mL RPMI-1640 plus 20% FBS was added to the lower compartment. The Transwell-containing plates were incubated for 24 h in the incubator. After incubation, cells that had migrated to the lower surface of the filter membrane were fixed with 4% paraformaldehyde for 25 min at room temperature. The membrane was washed 3 times with PBS and stained with 0.1% crystal violet in methanol for 15 min at room temperature. Cells remaining on the upper surface of the filter membrane (non-migrating) were gently scraped off with a cotton swab. The lower surfaces (with migrating cells) were captured by photomicroscopy (BX51 Olympus, Japan), and the cells were counted blindly (five fields per chamber).

### Immunofluorescence

SW480 cells were seeded on cell slide (Fisherbrand) in 24-well plates and then infected with miR-19a lentivirus or transfected with TIA1 vector. Two days later the cells were briefly washed with PBS, and then fixed in 4% paraformaldehyde for 30 min at room temperature (RT). After fixation, the cells were washed with PBS (3 × 5 min, RT), and then permeabilized and blocked using 5% BSA (Sigma) and 0.5% Triton X-100 in PBS for 1 h at RT. Next, the cells were incubated with primary antibody for Ki67 (Abcam, ab16667) in 5% BSA overnight at 4 °C, and then rinsed in PBS (3 × 5 min, RT). The cells were then incubated in secondary fluorescent antibody (Invitrogen, 594 nm) in 5% BSA in a light-proof environment for 1 h at RT. Next, the cells were stained with DAPI (Beyotime, Shanghai, China) a light-proof environment for 10 min at RT. Finally the cells were washed with PBS (3 × 5 min, RT) and visualized using a confocal microscope (C2+, Nikon).

### Colony formation assay

SW480 cells that were infected with miR-19a lentivirus or transfected with TIA1 vector were suspended in RPMI-1640 with 0.3% Agarose L.M.P (Biocam) solution, and plated onto solidified 0.6% Agarose L.M.P in 60 mm dish (Corning) at a density of 3 × 106 cells/mL in triplicate. The seeded cells were maintained in culture by feeding with 0.5 ml fresh RPMI-1640 plus 10% FBS and Penicillin-Streptomycin antibiotics (Gibco) every 3 days, for a total time 18 days. Then the colonies were fixed with 4% paraformaldehyde, and stained with 0.05% crystal violet. Then rinse the dish softly to scour off the redundant crystal violet. The shapes of colonies were capture by photomicroscopy (BX51 Olympus, Japan), and the numbers were counted by visual inspection.

### Establishment of tumor xenografts in mice

Four-week-old male SCID mice (*nu*/*nu*) were purchased from the Model Animal Research Center of Nanjing University (Nanjing, China) and maintained under specific pathogen-free conditions at Nanjing University. A lentiviral expression plasmid that can express miR-19a was purchased from GenePharma (Shanghai, China). Puromycin (Sigma-Aldrich, USA) was used to successfully obtain stably infected cells. SW480 cells were infected with a control lentivirus or a miR-19a lentivirus, transfected with a TIA1 overexpression plasmid, or co-transfected with a miR-19a overexpression lentivirus and a TIA1 overexpression plasmid. After infection and/or transfection, SW480 cells were subcutaneously injected into SCID mice (3 × 10^6^ cells in 0.2 mL PBS per mouse, 5 mice per group). The needle was inserted into the left side of the armpit, midway down, 5 mm deep at a 45° angle. Mice were sacrificed 24 days after injection to remove the xenografted tumors, and the volumes and weights of the tumors were measured. The volume of tumors were calculated by the formula: Tumor volume = [length * (width)^2^]/2 [35]. A portion of the tissues was used for protein and total RNA extraction, and the remaining tissue was fixed in 4% paraformaldehyde for 24 h. The tissue was processed for hematoxylin and eosin (H&E) staining or immunohistochemical staining for TIA1 and Ki-67. All experiments were approved by the Institutional Review Board of Nanjing University (Nanjing, China) and performed in accordance with the U.K. Animals (Scientific Procedures) Act (1986) and the guidelines of the National Institutes of Health.

### Statistical analysis

All of the images of the western blot assay, EdU assay, migration assay, immunofluorescence, colony formation or animal experiments were representative of at least three independent experiments or staining results. The quantitative RT-PCR assay, pull down assay, luciferase reporter assay, and proliferation assay were performed in triplicate, and each individual experiment was repeated several times. The results are presented as the means ± SE of at least three independent experiments. Observed differences were considered statistically significant at *p* < 0.05 by using Student’s *t*-test.

## Results

### TIA1 protein but not mRNA was downregulated in CRC tissues compared to normal adjacent tissues

**Fig. 1:**
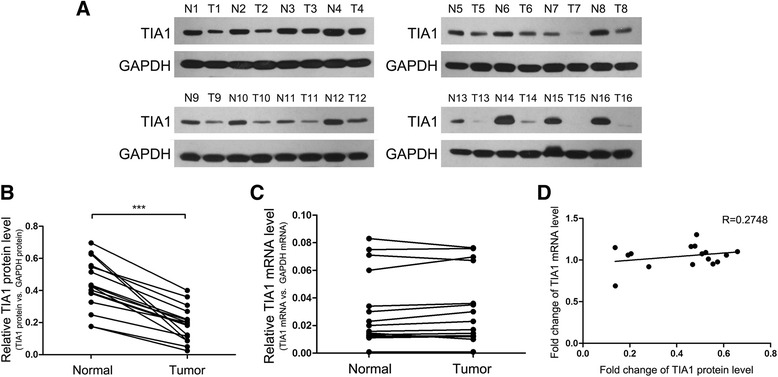
TIA1 protein but not mRNA was downregulated in CRC tissues compared to normal tissues. **a** and **b** Western blot analysis of TIA1 protein levels in 16 paired CRC (T) and normal adjacent tissue (N) samples. **a**: representative images; **b**: quantitative analysis. **c** Quantitative RT-PCR analysis of TIA1 mRNA levels in the same CRC and normal adjacent tissue sample pairs. **d** Pearson’s correlation scatter plot of the fold changes of TIA1 protein and mRNA levels in CRC tissue pairs. ***P < 0.001

First, we examined the expression pattern of TIA1 in human CRC tissues. In the 16 pairs of CRC tissues and normal adjacent tissues, we found that TIA1 protein levels were significantly decreased in CRC tissues compared to normal adjacent tissues (Fig. 1a and b). Similar TIA1 protein decreases were also observed in lung cancer (LC) tissues (Additional file 4: Figure S1A and B) and gastric cancer (GC) tissues (Additional file 4: Figure S1E and F). However, TIA1 mRNA levels displayed an irregular alteration (Fig. 1c). We then checked the relevance of the TIA1 protein and mRNA levels by using Pearson’s correlation scatter plots. The results showed that there was little correlation between the two levels (Fig. 1d). The inconsistency between TIA1 protein and mRNA expression in CRC tissues suggests that there may exist a post-transcriptional mechanism that downregulates the TIA1 protein levels in CRC tissues.

### Identification of TIA1 as a direct target gene of miR-19a

**Fig. 2:**
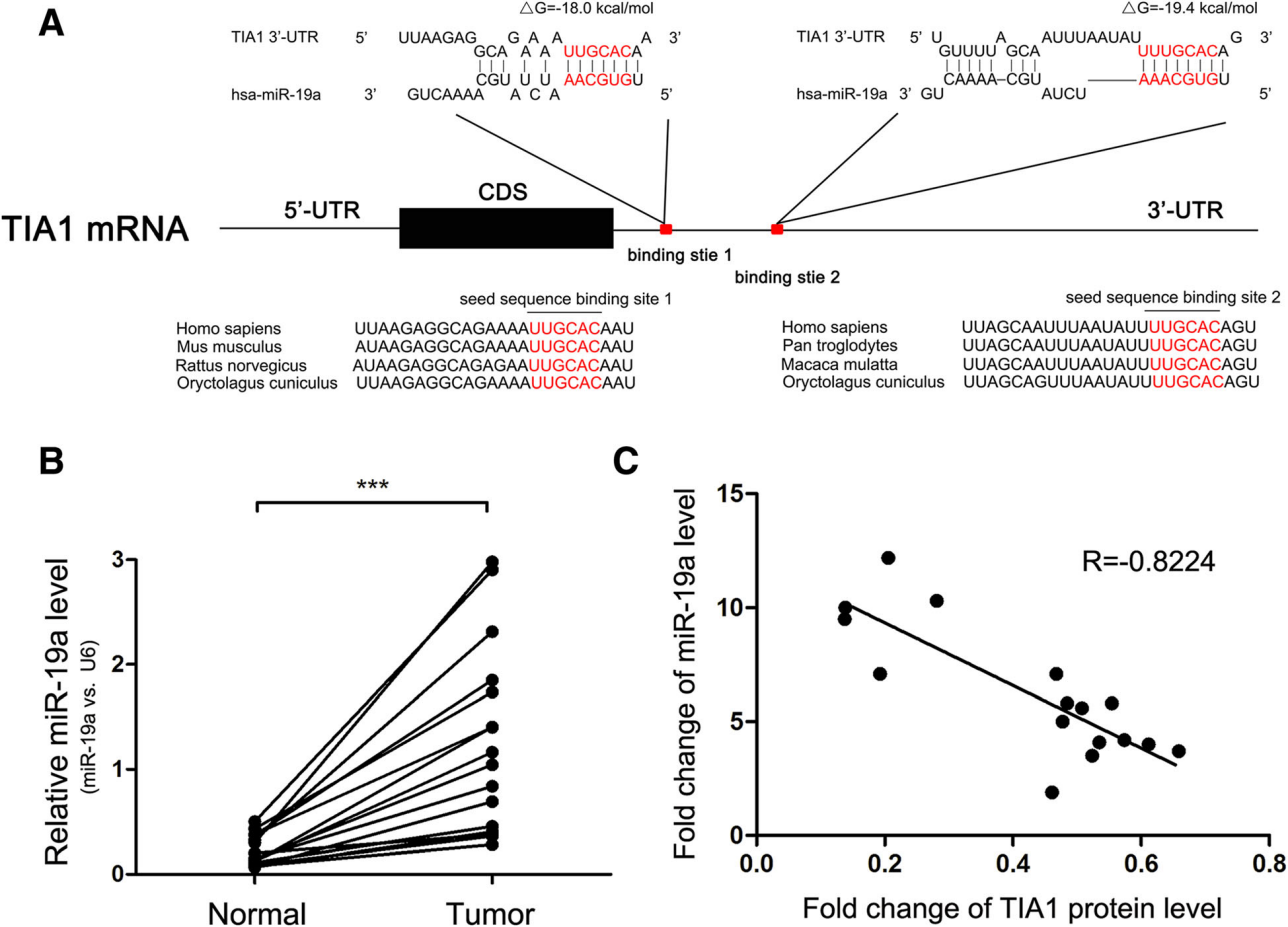
Identification of TIA1 as a miR-19a target. **a** Schematic description of the hypothetical duplex formed by the interactions between the binding site in the TIA1 3’-UTR and miR-19a. The miR-19a seed sequence and the seed sequence binding sites in the TIA1 3’-UTR are indicated in *red*. All nucleotides of the seed sequence of the binding site are conserved in several species, including human, mouse, rat and rabbit. The predicted free energy values of the hybrids are indicated. **b** Quantitative RT-PCR analysis of miR-19a expression levels in the same 16 pairs of CRC and normal tissue samples. **c** Pearson’s correlation scatter plot of the fold changes of miR-19a and TIA1 protein in human CRC tissue pairs. ***P < 0.001

Because miRNA is an important and widespread molecule that post-transcriptionally regulates gene expression, we hypothesized that TIA1 might be regulated by miRNA. Because miRNAs and their targets should have opposite expression pattern changes during tumorigenesis [36] and TIA1 protein levels were decreased in CRC tissues compared to normal tissues, we tried to identify miRNAs that are aberrantly increased during CRC transformation. To achieve this goal, we used an online database YM500 [37, 38] to perform a meta-analysis of the miRNA expression patterns of 8 normal solid tissues and 429 primary solid tumors of CRC. Among the 2578 miRNAs analyzed, 128 were found to be significantly upregulated and 145 were downregulated in the solid tumors compared to the normal tissues (fold change > 2 and *p*-value < 0.05) (Additional file 2: Table S2 and Additional file 5: Figure S2). Then, three computational algorithms, TargetScan [39], miRanda [40] and PicTar [41], were used in combination to investigate whether the aberrantly increased miRNAs could potentially target TIA1. Among the 10 most upregulated miRNAs (Additional file 3: Table S3), miR-19a and miR-19b (miR-19a/b) were identified as the candidate regulators of TIA1. miR-19a/b belong to the miR-19 family and only differ by a single nucleotide at position 11, a region minimally important for target recognition [42]. Therefore, miR-19a/b generally have overlapping target genes. The predicted interaction between miR-19a/b and the target sites in the TIA1 3’-UTR are illustrated in Fig. 2a and Additional file 6: Figure S3A. Two predicted hybridizations were identified between miR-19a/b and the 3’-UTR of TIA1. The minimum free energy values of the two hybridizations were -18.0 and -19.4 kcal/mol, and these values are well within the range of genuine miRNA-target pairs. Furthermore, the miR-19a/b binding sequences in the TIA1 3’-UTR are highly conserved across species (Fig. 2a and Additional file 6: Figure S3A).

We next investigated whether miR-19a/b expression levels were truly inversely correlated with TIA1 expression in CRC tissues. By measuring miR-19a/b levels in the same 16 pairs of CRC tissues and corresponding normal adjacent tissues, we found that miR-19a/b levels were consistently higher in CRC tissues (Fig. 2b and Additional file 6: Figure S3B). Furthermore, we illustrated the inverse correlation between miR-19a/b and TIA1 protein levels using Pearson’s correlation scatter plots. As shown in Fig. 2c and Additional file 6: Figure S3C, miR-19a expression has a higher inverse correlation coefficient (*R* = -0.8224) with TIA1 expression than miR-19b (*R* = -0.6425). Thus, although miR-19a/b have similar sequences, similar expression patterns and the same binding potential to TIA1, we focused on miR-19a for further experiments. Then we measured miR-19a level in LC and GC tissues mentioned above, and we also observed upregulated miR-19a level and reverse correlation between miR-19a and TIA1 protein levels in the two cancer types (Additional file 4: Figure S1C, D, G and H).

### miR-19a directly regulates TIA1 expression at the post-transcriptional level

**Fig. 3:**
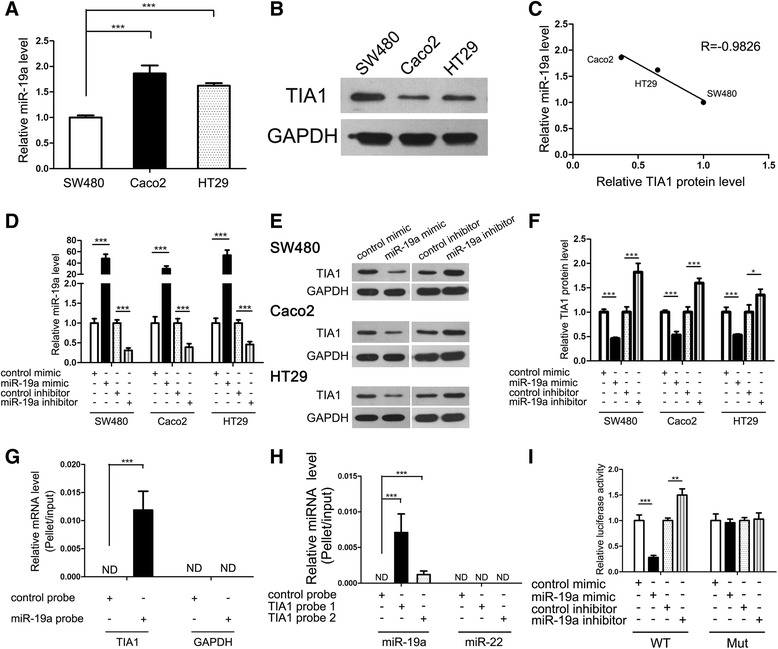
miR-19a directly regulates TIA1 expression at the post-transcriptional level. **a** Quantitative RT-PCR analysis of miR-19 levels in SW480, Caco2 and HT-29 cells. miR-19 level of SW480 was set as control. **b** Western blot analysis of TIA1 protein levels in SW480, Caco2 and HT-29 cells. TIA1 level of SW480 was set as control. **c** Pearson’s correlation scatter plot of the level of miR-19 and TIA1 protein in SW480, Caco2 and HT-29 cells. **d** Quantitative RT-PCR analysis of miR-19a levels in SW480, Caco2 and HT-29 cells transfected with control mimic, miR-19a mimic, control inhibitor or miR-19a inhibitor. **e** and **f** Western blot analysis of TIA1 protein levels in SW480, Caco2 and HT-29 cells transfected with control mimic, miR-19a mimic, control inhibitor or miR-19a inhibitor. **e**: representative images; **f**: quantitative analysis. **g** Quantitative RT-PCR analysis of TIA1 and GAPDH mRNA levels in SW480 after pulling down with control probe or miR-19a probe; **h** Quantitative RT-PCR analysis of miR-19a and miR-22 levels in SW480 after pulling down with control probes or two TIA1 mRNA probes; **i** The relative luciferase activities in SW480 transfected with wild type or mutant TIA1 3’-UTR. *P < 0.05; **P < 0.01; ***P < 0.001

We modulated miR-19a levels in CRC cell lines to investigate whether TIA1 was inversely correlated with miR-19a in vitro. First, we measured miR-19a and TIA1 protein levels in three CRC cell lines, SW480, Caco2 and HT29. As expected, an inverse correlation between miR-19a and TIA1 expression was observed in these CRC cell lines (Fig. 3a–c). Next, we efficiently overexpressed or knocked down the miR-19a level in SW480 cells with a miR-19a mimic or inhibitor, respectively (Fig. 3d). As anticipated, TIA1 protein levels dramatically decreased upon miR-19a overexpression, whereas treatment with the miR-19a inhibitor increased TIA1 protein levels (Fig. 3e and f). However, the alteration of miR-19a had little effect on the TIA1 mRNA level (Additional file 7: Figure S4A). Moreover, we repeated the above transfection experiments in two other CRC cell lines (Caco2 and HT29), one LC cell line (A549) and one GC cell line (MKN) to validate the robustness of the test. miR-19a repressed TIA1 expression in all the four cells, too (Fig. 3d–f, Additional file 4: Figure S1I and J and Additional file 7: Figure S4A).

To validate that miR-19a suppressed TIA1 expression through direct interaction with the binding site in the TIA1 3’-UTR, we did pull down assay and luciferase reporter assay. Firstly, we performed a biotin-avidin pull-down assay to assess the direct binding of miR-19a to TIA1 mRNA. We designed a miR-19a mimic whose 3’ terminal was biotinylated (miR-19a probe). Such modification did not affect the suppression of TIA1 by miR-19a (Additional file 7: Figure S4B and C). After transfecting miR-19a probe into SW480 for 48 h, we used streptavidin-coated magnetic beads to pull down biotinylated miR-19a and measured the co-precipitated TIA1 mRNA. TIA1 mRNA was only enriched in the pull-down product precipitated by the miR-19a probe and was undetectable in the product that was precipitated by control probe (Fig. 3g), suggesting that miR-19a directly binds to TIA1 mRNA in SW480 cells. As a control, GAPDH mRNA could not be detected in the pull-down product precipitated by the miR-19a probe (Fig. 3g), since GAPDH was not a predicted target of miR-19a according to the target prediction softwares. Furthermore, we used two biotinylated anti-TIA1 mRNA probes and streptavidin-coated magnetic beads to pull down TIA1 mRNA fragments and measured the co-precipitated miR-19a. Anti-TIA1 mRNA probes efficiently precipitated TIA1 mRNA fragments, as significant enrichment of TIA1 mRNA fragments was detected in the pull-down products precipitated by the anti-TIA1 mRNA probes (Additional file 7: Figure S4D). Consequently, miR-19a was only enriched in the pull-down products precipitated by the anti-TIA1 mRNA probes and was unot present in the product precipitated by control probe (Fig. 3h). As a control, miR-22 could not be detected in the pull-down products precipitated by the anti-TIA1 mRNA probes (Fig. 3h), since miR-22 was not supposed to bind TIA1 according to the target prediction software.

To check if it was the predicted seed sequence binding sites that caused the miRNA-mRNA interaction, we constructed a firefly reporter plasmid containing a fragment of TIA1 3’-UTR across the two conserved miR-19a binding sites and then transfected the resulting plasmid into SW480 cells along with the miR-19a mimic, miR-19a inhibitor or scrambled negative control RNAs. As expected, miR-19a overexpression resulted in an approximately 70% reduction in luciferase reporter activity compared to cells transfected with the control mimic, whereas miR-19a inhibition resulted in a 50% increase in reporter activity compared to cells transfected with the control inhibitor (Fig. 3i). Subsequently, we mutated the miR-19a binding sites in the TIA1 3’-UTR fragment on the reporter plasmid to eliminate miR-19a binding ability. miR-19a overexpression or knockdown no longer affected the mutated reporter activity (Fig. 3i), suggesting that the binding sites strongly contribute to the miRNA-mRNA interactions.

### c-MYC, miR-19a, TIA1 and PDCD4 form a regulatory axis in CRC

**Fig. 4:**
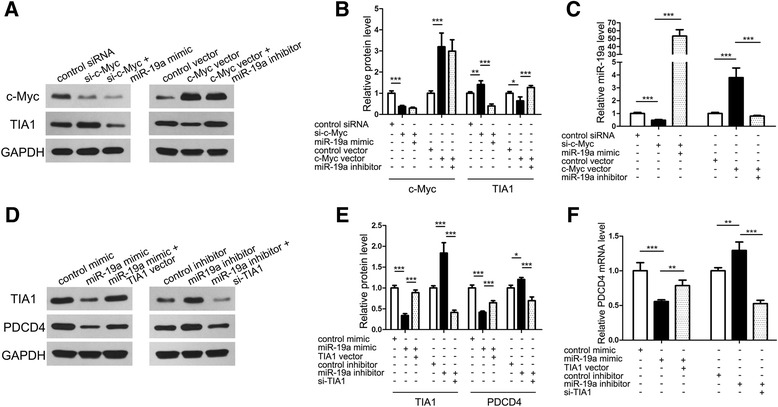
c-MYC, miR-19a, TIA1 and PDCD4 form a regulatory axis in CRC. **a** and **b** Western blot analysis of c-Myc and TIA1 protein levels in SW480 cells transfected with control siRNA, si-c-Myc, si-c-Myc plus miR-19a mimic, control plasmid, c-Myc plasmid, or c-Myc plasmid plus miR-19a inhibitor. **a**: representative images; **b**: quantitative analysis. **c** Quantitative RT-PCR analysis of miR-19a levels in SW480 cells transfected with control siRNA, si-c-Myc, si-c-Myc plus miR-19a mimic, control plasmid, c-Myc plasmid, or c-Myc plasmid plus miR-19a inhibitor. **d** and **e** Western blot analysis of TIA1 and PDCD4 protein levels in SW480 cells transfected with control mimic, miR-19a mimic, miR-19a mimic plus TIA1 plasmid, control inhibitor, miR-19a inhibitor, or miR-19a inhibitor plus si-TIA1. **d**: representative images; **e**: quantitative analysis. **f** Quantitative RT-PCR analysis of PDCD4 mRNA levels in SW480 cells transfected with control mimic, miR-19a mimic, miR-19a mimic plus TIA1 plasmid, control inhibitor, miR-19a inhibitor, or miR-19a inhibitor plus si-TIA1. ND: Non-Detectable. *P < 0.05; **P < 0.01; ***P < 0.001

As one of the most important oncomiR clusters, the miR-17-92 cluster is regulated by many upstream transcriptional factors [43]. The best-studied of these factors is c-Myc [29, 44, 45]. Because c-Myc can transcriptionally upregulate miR-19a, we speculated that c-Myc also influences the miR-19a target gene TIA1. We used siRNA or an overexpression plasmid to silence or enhance c-Myc expression in SW480 cells, respectively (Fig. 4a and b). We observed the anticipated decrease or increase in miR-19a levels after treatment with c-Myc siRNA or the overexpression plasmid, respectively (Fig. 4c). As a consequence, TIA1 protein levels showed an inverse alteration trend compared to the expression of c-Myc and miR-19a (Fig. 4a and b). We then transfected SW480 cells with the miR-19a mimic or inhibitor to reverse the effect of the c-Myc siRNA or overexpression plasmid on miR-19a, respectively (Fig. 4c). As expected, TIA1 protein expression was attenuated by co-treatment with the miR-19a mimic and c-Myc siRNA compared with treatment with c-Myc siRNA alone. In contrast, reduced TIA1 protein expression caused by the c-Myc overexpression plasmid was rescued by the co-added miR-19a inhibitor (Fig. 4a and b). To exclude the possibility that c-Myc directly suppressed TIA1 at the transcriptional level, we measured the TIA1 mRNA level after treatment with the c-Myc siRNA or overexpression plasmid and detected no significant alteration in the TIA1 mRNA level (Additional file 8: Figure S5A).

TIA1 is an important gene expression modulator, especially in cancers [10]. We proposed that, by suppressing TIA1, miR-19a also indirectly influences its myriad downstream targets. PDCD4, an important tumor suppressor in CRC [9], has been reported to be regulated by TIA1 [46]. We chose PDCD4 as a target to investigate whether miR-19a modulates PDCD4 expression through regulation of TIA1. First, we screened the full PDCD4 mRNA (including 5’-UTR, CDS and 3’-UTR) and identified no miR-19a binding site. Then, we repeated the miR-19a mimic and inhibitor transfection experiment in SW480 cells and measured both the TIA1 and PDCD4 protein levels. PDCD4 displayed the same trend in the alteration in the direction of the TIA1 protein after treatment with the miR-19a mimic or inhibitor (Fig. 4d and e).

When the TIA1 overexpression plasmid or siRNA (the efficacies of which were shown in Additional file 8: Figure S5B–D) was used to neutralize the effects of the miR-19a mimic or inhibitor on TIA1 expression, respectively, the PDCD4 levels were also recovered (Fig. 4d and e). We measured the influence of miR-19a and TIA1 on PDCD4 mRNA levels and verified that miR-19a and TIA1 affected PDCD4 at the transcriptional level (Fig. 4f). Taken together, the results reveal that there is a c-MYC-miR-19a-TIA1-PDCD4 regulatory axis in CRC cells.

### miR-19a promotes CRC cell proliferation by targeting TIA1

**Fig. 5:**
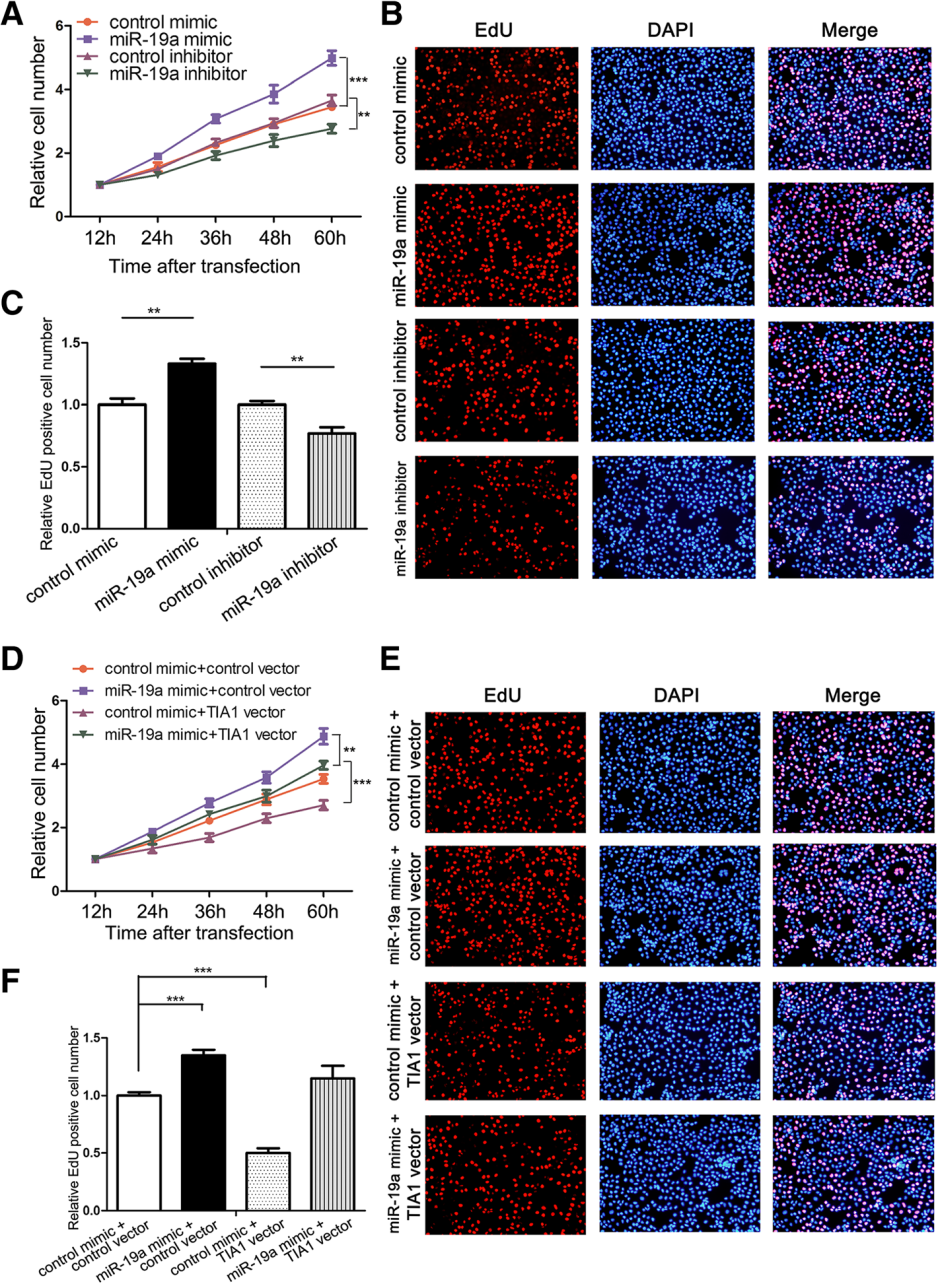
miR-19a promotes CRC cell proliferation by targeting TIA1. **a** Cell proliferation assays (CCK-8) were performed 12, 24, 36, 48 and 60 h after the transfection of SW480 cells with equal doses of control mimic, miR-19a mimic, control inhibitor or miR-19a inhibitor. **b** and **c** Cell proliferation assays (EdU) were performed in SW480 cells transfected with equal doses of control mimic, miR-19a mimic, control inhibitor or miR-19a inhibitor. **b**: representative images; **c**: quantitative analysis. **d** Cell proliferation assays (CCK-8) were performed 12, 24, 36 and 48 and 60 h after transfection of SW480 cells with equal doses of control mimic plus control plasmid, miR-19a mimic plus control plasmid, control mimic plus TIA1 overexpression plasmid, or miR-19a mimic plus TIA1 overexpression plasmid. **e** and **f** Cell proliferation assays (EdU) were performed in SW480 cells transfected with equal doses of control mimic plus control plasmid, miR-19a mimic plus control plasmid, control mimic plus TIA1 overexpression plasmid, or miR-19a mimic plus TIA1 overexpression plasmid. **e**: representative images; **f**: quantitative analysis. **P < 0.01; ***P < 0.001

We hypothesized that miR-19a can promote CRC progression by suppressing TIA1. Thus, we performed CCK-8 and EdU assays to investigate the effect of miR-19a on CRC cell proliferation. SW480 cells transfected with a miR-19a mimic exhibited increased proliferation; in contrast, miR-19a inhibition had the opposite effect on cell proliferation (Fig. 5a–c). Because a single miRNA has multiple target genes [36], it is necessary to determine whether the effect of miR-19a on CRC cell proliferation is derived from miR-19a-mediated TIA1 suppression. We therefore investigated the exact contribution of the miR-19a-TIA1 axis on CRC cell proliferation. We used TIA1 siRNA or an overexpression plasmid to silence or enhance TIA1 protein expression, respectively. As a result, TIA1 knockdown activated the proliferation of SW480 cells, whereas TIA1 overexpression had the opposite effect on cell proliferation (Additional file 9: Figure S6A–D). The results indicated that miR-19a and TIA1 have opposite effects on CRC cell proliferation. We then investigated whether rescuing miR-19a-mediated-TIA1 suppression with a TIA1 overexpression plasmid attenuates the pro-proliferation effect of miR-19a on CRC cells. As expected, cells co-transfected with the miR-19a mimic and TIA1 overexpression plasmid showed lower proliferation rates compared to cells transfected with the miR-19a mimic alone (Fig. 5d–f). Thus, restoration of TIA1 expression can reverse miR-19a-induced cell proliferation, suggesting that targeting TIA1 is one mechanism by which miR-19a exerts its oncomiR function.

### miR-19a promotes CRC cell migration by targeting TIA1

**Fig. 6:**
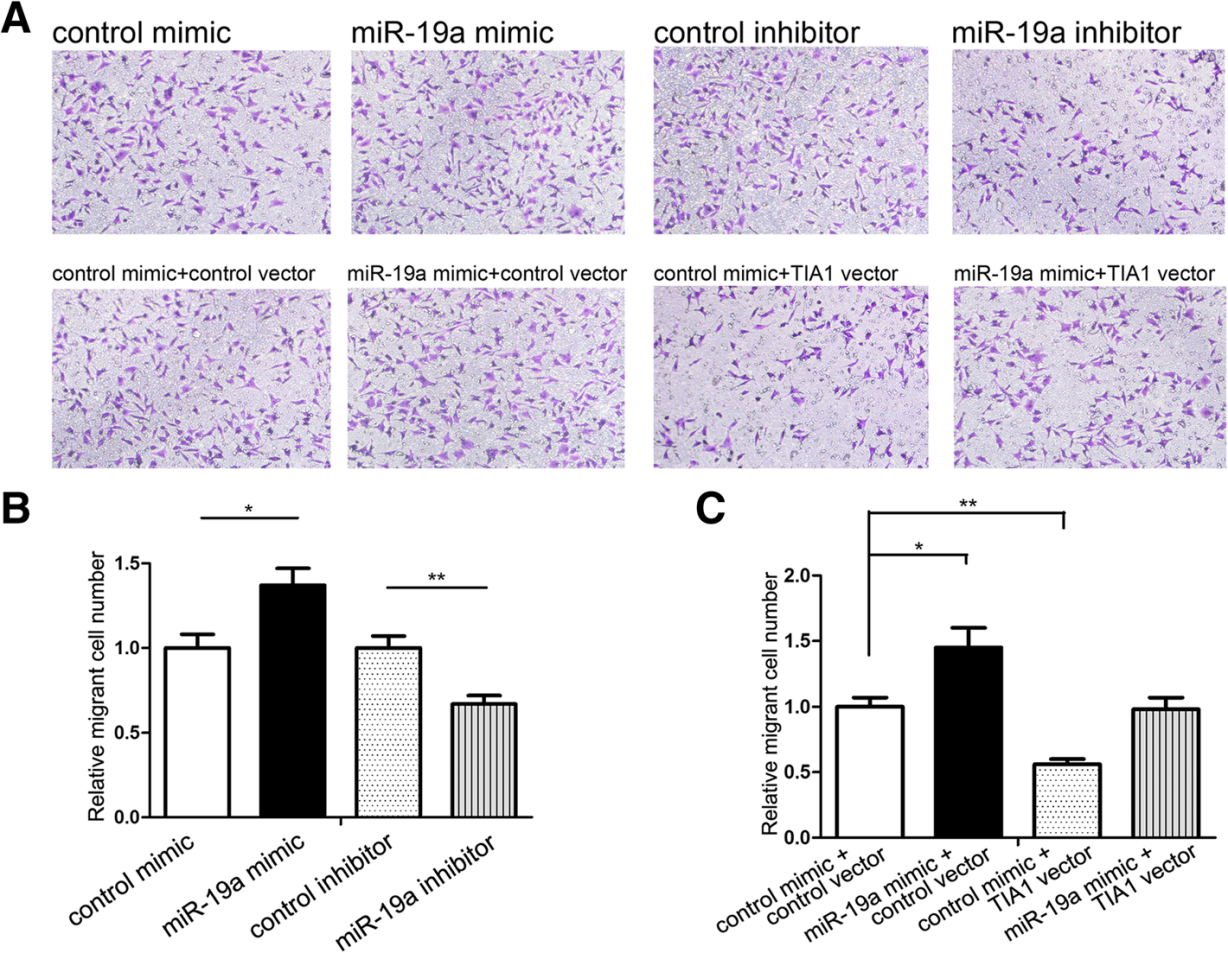
miR-19a promotes CRC cell migration by targeting TIA1. **a**–**c** Cell migration assays (Transwell) were performed in SW480 cells that were transfected with equal doses of control mimic, miR-19a mimic, control inhibitor or miR-19a inhibitor (upper panel), or with equal doses of control mimic plus control plasmid, miR-19a mimic plus control plasmid, control mimic plus TIA1 overexpression plasmid, or miR-19a mimic plus TIA1 overexpression plasmid (lower panel). **a**: representative images; **b** and **c**: quantitative analysis of the number of migrated cells. *P < 0.05; **P < 0.01

Next, we performed a Transwell assay to investigate the effect of miR-19a on CRC cell migration. Similar to the effect on SW480 proliferation, miR-19a promoted SW480 migration (Fig. 6a and b). We then validated that the promotion of cell migration by miR-19a was a result of the inhibition of TIA1. SW480 cells transfected with TIA1 siRNA showed increased cell migration; in contrast, transfection with the TIA1 overexpression plasmid had the opposite effect on cell migration (Additional file 9: Figure S6E and F). Moreover, compared with cells transfected with miR-19a mimic alone, those transfected with both the miR-19a mimic and TIA1 overexpression plasmid exhibited a significantly lower migration rate (Fig. 6a and c), suggesting that miR-19a-resistant TIA1 is sufficient to rescue the suppression of TIA1 through miR-19a and attenuate the pro-migration effect of miR-19a on CRC cells. Taken together, these data revealed that miR-19a promotes CRC cell migration by silencing TIA1.

### miR-19a promotes CRC growth in vivo by targeting TIA1

**Fig. 7:**
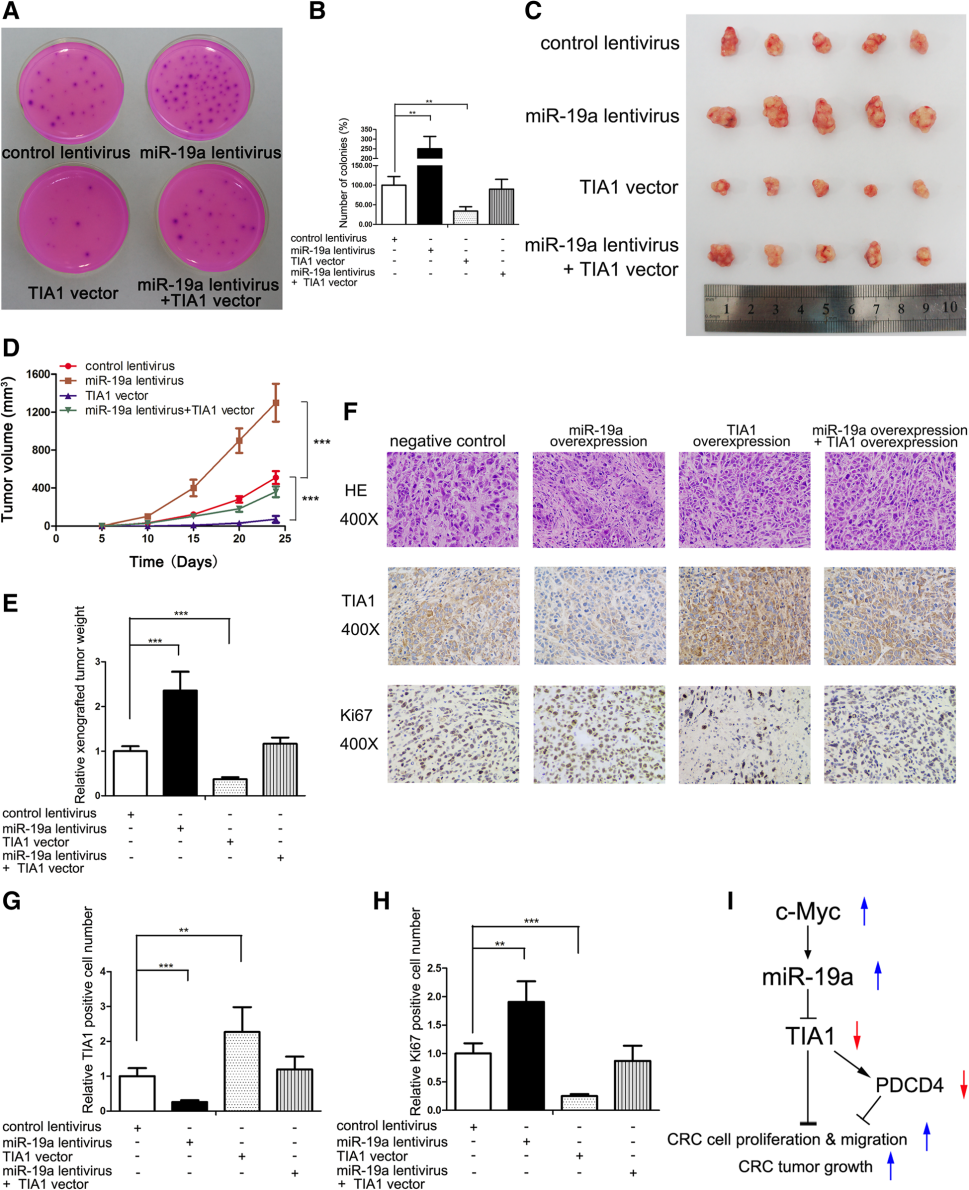
miR-19a promotes CRC growth in vitro and in vivo by targeting TIA1. **a** and **b** Colony formation assays of SW480 cells infected with miR-19a lentivirus, transfected with TIA1 vector or both. **a**: Representative images of colony formation assays; **b**: quantitative analysis of the numbers of colonies. **c** Representative images of tumors from the implanted mice. **d** and **e** Quantitative analysis of xenografted tumor volume and weight. **d**: tumor volume; **e**: tumor weight. **f**–**h** H&E-stained sections and immunohistochemical staining for TIA1 and Ki-67 in tumors from implanted mice. **f**: representative images; **g** and **h**: quantitative analysis. **i** Working model of the c-Myc-miR-19a-TIA1-PDCD4 regulatory axis in CRC. **P < 0.01; ***P < 0.001

Finally, we investigated the effects of miR-19a and TIA1 on the growth of CRC xenografts in nude mice. To prepare the nude mouse xenograft model, we first infected SW480 cells with a miR-19a overexpression lentivirus, transfected SW480 cells with a TIA1 overexpression plasmid, or co-transfected SW480 cells with the miR-19a overexpression lentivirus and TIA1 overexpression plasmid. The upregulation of miR-19a and consequential suppression of TIA1 protein by lentivirus infection are shown in Additional file 10: Figure S7A–C. We then examined the effect of miR-19a lentivirus or TIA1 vector on SW480 proliferation and malignancy. Ki-67 immunofluorescence and soft-agar colony formation assay results indicated that miR-19a could promote SW480 cells proliferation and anchorage-independent growth by targeting TIA1 (Fig. 7a and b, Additional file 10: Figure S7D–F). Next, we subcutaneously implanted the infected or transfected SW480 cells into 4-week-old nude mice. We evaluated tumor growth 24 days after cell implantation. The volumes or weights of xenografted tumors were smaller or lower in the control group than in the miR-19a-overexpressing group but larger or higher than in the TIA1-overexpressing group, respectively (Fig. 7c–e). The overexpression of TIA1 attenuated the growth-promoting effects of miR-19a, suggesting that miR-19a promotes tumor growth by silencing TIA1 (Fig. 7c–e). We next isolated and analyzed total RNA and protein from the tumors. Significant upregulation of miR-19a or TIA1 was observed in the miR-19a-overexpressing or TIA1-overexpressing groups, respectively, and TIA1 overexpression could truly reverse the inhibition effect of miR-19a on TIA1 (Additional file 10: Figure S7G–I). We then embedded xenografted tumors in paraffin and performed H&E staining or examined the tumors using immunohistochemical assays. H&E results showed increased cell mitosis in the miR-19a-overexpressing group and decreased mitosis in the TIA1-overexpressing group, whereas xenografts with both miR-19a and TIA1 overexpression exhibited less cell mitosis compared to xenografts with miR-19a overexpression alone (Fig. 7f). Immunohistochemical staining revealed lower TIA1 and higher Ki-67 levels in tumors from the miR-19a-overexpressing group, whereas tumors from the TIA1-overexpressing group showed increased TIA1 and decreased Ki-67 levels (Fig. 7f–h). TIA1 overexpression in the miR-19a lentivirus-infected group restored the suppression of TIA1 and Ki-67 caused by miR-19a overexpression (Fig. 7f–h). These results are consistent with the in vitro findings, which firmly validated the oncomiR role of miR-19a in CRC tumorigenesis through targeting TIA1.

## Discussion

In 2016, the American Cancer Society estimated that there would be more than 130,000 new cases of and more than 49,000 new deaths from CRC in the United States, making it the third leading cause of death from cancer [1]. Although recent advances in medicine have improved the survival of CRC patients, CRC remains a major public health problem. The clinical behavior of colorectal cancer results from interactions at many levels [2], and among these interactions, the genetic changes in oncogenes and/or tumor-suppressor genes are the most important driving force for tumorigenesis and cancer development. TIA1 is an important tumor suppressor in many cancers [10], especially in CRC [15, 16]. In this study, we demonstrated that TIA1 protein was significantly downregulated in CRC tissues and inhibited proliferation and migration of CRC cells and attenuated tumor growth in xenografted mice. Interestingly, we observed that TIA1 mRNA levels did not exhibit alterations consistent with the protein levels. This phenomenon inspired us to speculate that a post-transcriptional mechanism is involved in the repression of TIA1 expression in CRC. Therefore, we focused on a vital post-transcriptional regulator, miRNA, and provided the first evidence supporting the important role of miR-19a as an oncomiR in CRC targeting TIA1.

The discovery of miRNAs expanded the list of classical tumor suppressors and oncogenes. miRNA profiles undergo a wide array of alterations during the initiation and development of various types of cancer, including CRC [47–49]. More importantly, new studies have established the potential usefulness of miRNAs as therapeutic molecules against cancer [50–53]. For example, aberrantly dysregulated miRNAs can be restored by using antagomirs or miRNA mimics [50, 53]. miR-19a belongs to a well-known oncomiR cluster, namely the miR-17-92 cluster. miR-19a has been widely studied and is thought to be a key oncogenic component of mir-17-92 [54]. miR-19a is overexpressed and functions as an oncomiR in many cancer types, including bladder cancer [55], cervical cancer [56], gastric cancer [57], pancreatic cancer [58], renal cancer [59], lung cancer [60] and CRC [32–34]. In this study, we performed a meta-analysis of the miRNA expression profile in CRC and found that some miRNAs (especially miR-19a) were dramatically upregulated. We then used 3 bioinformatic algorithms to predict miRNAs that could target TIA1 and identified miR-19a as an ideal candidate. Subsequently, we experimentally validated TIA1 as a genuine miR-19a target in three CRC cell lines. Furthermore, we revealed the important effects of miR-19a-driven suppression of TIA1 on the promotion of CRC cell proliferation and migration and on tumor growth in a xenografted nude mouse model. These results suggested that targeting miR-19a may be a practical method to control CRC development and ameliorate symptoms, as has been shown by other groups [50, 51, 53]. More research should focus on characterizing the feasibility of targeting miR-19a in CRC therapy and developing simplified and cost-effective manipulation methods.

The c-Myc oncogene contributes to the genesis of many human cancers [61, 62], including CRC [63]. c-Myc functions as a transcriptional activator or inhibitor that modulates almost every aspect of tumor cell biology [64]. In recent years, the ability of c-Myc to regulate miRNAs has received much attention [65]. Many of the functions of c-Myc in cancers are executed through its downstream miRNAs [66]. Among the miRNA targets of c-Myc, the miR-17-92 cluster is the most well-studied [29, 44, 45]. c-Myc has been shown to upregulate the expression of the mir-17-92 cluster to accelerate tumor development in a mouse B-cell lymphoma model [29]. Increased expression of the miR-17-92 cluster during colorectal adenoma to adenocarcinoma progression is also associated with c-Myc expression [45]. Interestingly, c-Myc influences its target miRNAs to indirectly regulate the target genes of the miRNAs. For example, the c-Myc-regulated miR-17-92 cluster can modulate E2F1 expression to promote cell cycle progression [44]. In this study, we showed that c-Myc indirectly suppresses TIA1 expression through enhancing miR-19a expression in CRC. These findings provide new insights into understanding how c-Myc contributes to CRC development. In addition, another member of the TIA family, T-cell intracellular antigen 1 (TIA1)-related protein (TIAR), inhibits translation of c-Myc mRNA via AU-rich elements [67]. This finding, combined with ours, reveals the complex relationship between c-Myc and TIA family.

As an important gene expression modulator, TIA1 regulates many downstream genes [10]. Under conditions of rapid oxygen decline and extreme hypoxia, TIA1 co-aggregates with TIAR to suppress the HIF-1α pathway [68]. TIA1 can also function as a translational silencer of COX-2 expression in neoplasia [69]. Moreover, TIA1 even affects the expression of some miRNAs [70]. It is easy to speculate that by suppressing TIA1, miR-19a may also influence the downstream genes of TIA1. In this study, we proved that miR-19a inhibits TIA1 to indirectly downregulate PDCD4, which is a TIA1 target [46] as well as an important tumor suppressor [9] in CRC. Some oncomiR functions of miR-19a may be indirectly executed by inhibiting its indirect target PDCD4. Thus, TIA1 functions as a bridge to link upstream regulators (such as miR-19a) and downstream effectors (such as PDCD4). We suppose that there are many other indirect regulation relationships similar to miR-19a and PDCD4, and these relationships greatly complicate the miR-19a—target regulatory network.

## Conclusions

In summary, this study demonstrated for the first time that miR-19a can target TIA1 to promote CRC tumorigenesis, and we identified the c-MYC—miR-19a—TIA1—PDCD4 axis in CRC (The working model was shown in Fig. 7i). More research should be conducted on miR-19a and TIA1, and we would like to further elucidate the molecular mechanisms of CRC and develop new approaches for molecular therapeutics for this disease.

## Additional files

Additional file 1: Table S1. Clinical features of colorectal cancer patients. (DOCX 17 kb)
Additional file 2: Table S2. Detailed information on 273 significantly changed miRNAs in 429 primary solid colon tumors and 8 normal solid tissues from a meta-analysis using YM500. (XLSX 47 kb)
Additional file 3: Table S3. Top 29 significantly upregulated miRNAs in the colon cancer tissues compared with the normal solid tissues analyzed by meta-analysis by using YM500. (DOCX 17 kb)
Additional file 4: Figure S1. miR-19a can also target TIA1 in LC and GC tissues and cell lines. (A and B) Western blot analysis of TIA1 protein levels in 8 paired LC (T) and normal adjacent tissue (N) samples. A: representative images; B: quantitative analysis. (C) Quantitative RT-PCR analysis of miR-19a levels in the same LC and normal adjacent tissue sample pairs. (D) Pearson’s correlation scatter plot of the fold changes of TIA1 protein and miR-19a levels in LC tissue pairs. (E and F) Western blot analysis of TIA1 protein levels in 11 paired GC (T) and normal adjacent tissue (N) samples. E: representative images; F: quantitative analysis. (G) Quantitative RT-PCR analysis of miR-19a levels in the same GC and normal adjacent tissue sample pairs. (H) Pearson’s correlation scatter plot of the fold changes of TIA1 protein and miR-19a levels in GC tissue pairs. (I and J) Western blot analysis of TIA1 protein levels in A549 and MKN cells transfected with control mimic, miR-19a mimic, control inhibitor or miR-19a inhibitor. E: representative images; F: quantitative analysis. **P* < 0.05; ***P* < 0.01; ****P* < 0.001. (TIF 1406 kb)
Additional file 5: Figure S2. Profiles of 273 significantly changed miRNAs in 429 primary solid colon tumors and 8 normal solid tissues from a meta-analysis by using YM500. Detailed information on these miRNAs is listed in Additional file 2: Table S2. (TIF 1762 kb)
Additional file 6: Figure S3. miR-19b can also regulate TIA1 expression in CRC. (A) Schematic description of the hypothetical duplex formed by the interaction between the binding site in the TIA1 3’-UTR and miR-19b. The miR-19b seed sequence and the seed sequence binding sites in the TIA1 3’-UTR are indicated in red. All nucleotides of the seed sequence of the binding site are conserved in several species, including human, mouse, rat and rabbit. The predicted free energy values of the hybrids are indicated. (B) Quantitative RT-PCR analysis of miR-19b expression levels in the same 16 pairs of CRC and normal tissue samples. (C) Pearson’s correlation scatter plot of the fold change of miR-19b and TIA1 protein in human CRC tissue pairs. (D) Quantitative RT-PCR analysis of miR-19b levels in SW480 cells transfected with control mimic, miR-19b mimic, control inhibitor or miR-19b inhibitor. (E and F) Western blot analysis of TIA1 protein levels in SW480 cells transfected with control mimic, miR-19b mimic, control inhibitor or miR-19b inhibitor. E: representative images; F: quantitative analysis. ****P* < 0.001. (TIF 917 kb)
Additional file 7: Figure S4. Effect of miR-19a on TIA1 mRNA level and the efficacies of miR-19a probe and TIA1 mRNA probes. (A) Quantitative RT-PCR analysis of TIA1 mRNA levels in SW480, Caco2 and HT-29 cells transfected with control mimic, miR-19a mimic, control inhibitor or miR-19a inhibitor. (B and C) Western blot analysis of TIA1 protein levels in SW480 cells transfected with control mimic, miR-19a mimic or biotinylated miR-19a mimic. B: representative images; C: quantitative analysis. (D) Quantitative RT-PCR analysis of TIA1 and GAPDH mRNA levels in SW480 after pulling down with control probe or TIA1 mRNA probes. ***P* < 0.01; ****P* < 0.001. (TIF 371 kb)
Additional file 8: Figure S5. Effect of c-Myc on TIA1 mRNA level and the efficacies of TIA1 siRNA and overexpression vector. (A) Quantitative RT-PCR analysis of TIA1 mRNA levels in SW480 cells transfected with control siRNA, si-c-Myc, control plasmid or c-Myc plasmid. (B and C) Western blot analysis of TIA1 protein levels in SW480 cells transfected with control siRNA, si-TIA1, control vector or TIA1 vector. B: representative images; C: quantitative analysis. (D) Quantitative RT-PCR analysis of TIA1 mRNA levels in SW480 cells transfected with control siRNA, si-TIA1, control vector or TIA1 vector. ****P* < 0.001. (TIF 602 kb)
Additional file 9: Figure S6. Effects of TIA1 on SW480 proliferation and migration. (A and B) Cell proliferation assays (CCK-8) were performed 12, 24, 36, 48 and 60 h after the transfection of SW480 cells with equal doses of control siRNA, si-TIA1, control plasmid or TIA1 plasmid. (C and D) Cell proliferation assays (EdU) were performed in SW480 cells transfected with equal doses of control siRNA, si-TIA1, control plasmid or TIA1 plasmid. C: representative images; D: quantitative analysis. (E and F) Cell migration assays (transwell) were performed in SW480 cells that were transfected with equal doses of control siRNA, si-TIA1, control plasmid or TIA1 plasmid. E: representative images; F: quantitative analysis. **P* < 0.05; ***P* < 0.01; ****P* < 0.001. (TIF 4077 kb)
Additional file 10: Figure S7. The efficiency of miR-19a lentivirus and TIA1 vector on TIA1 protein level and SW480 proliferation. (A) Quantitative RT-PCR analysis of miR-19a levels in SW480 cells, which were infected with a control lentivirus or a lentivirus to overexpress miR-19a. (B and C) Western blot analysis of TIA1 protein levels in SW480 cells, which were infected with a control lentivirus or a lentivirus to overexpress miR-19a. B: representative images; C: quantitative analysis. (D-F) miR-19a could promote SW480 cell proliferation by targeting TIA1 in vitro. D representative images of Ki67 immunofluorescence; E: quantitative analysis of Ki67 immunofluorescence; F: representative images of formed SW480 colonies. (G) Quantitative RT-PCR analysis of miR-19a levels in tumors from implanted mice. (H and I) Western blot analysis of TIA1 protein levels in tumors from implanted mice. H: representative images; I: quantitative analysis. ***P* < 0.01; ****P* < 0.001. (TIF 3290 kb)

## Abbreviations

3’-UTR: 3’-untranslated region
5’-UTR: 5’-untranslated region
CDS: Coding sequence
CRC: Colorectal cancer
GC: Gastric cancer
LC: Lung cancer
miRNA: microRNA
PDCD4: Programmed cell death 4
TIA1: T-cell intracellular antigen 1
TIAR: T-cell intracellular antigen 1 (TIA1)-related protein
