# Supplementary material for: miR-19a promotes colorectal cancer proliferation and migration by targeting TIA1
Source: Mol Cancer. 2017 Mar 4;16:53. doi: 10.1186/s12943-017-0625-8 (PMC5336638; doi:10.1186/s12943-017-0625-8)
Supplement: Additional file 1: Table S1. — Clinical features of colorectal cancer patients. (DOCX 17 kb) [file 12943_2017_625_MOESM1_ESM.docx]

**miR-19a promotes colorectal cancer proliferation and migration by targeting TIA1**

**Supplemental Table 1. Clinical features of colorectal cancer patients.**

| Case number | Age | Gender | TNM stage | Cancer subtype |
| --- | --- | --- | --- | --- |
| 1 | 47 | F | III | colon carcinoma |
| 2 | 65 | M | III | colon carcinoma |
| 3 | 53 | F | III | colon carcinoma |
| 4 | 64 | M | III | rectal carcinoma |
| 5 | 56 | M | IV | colon carcinoma |
| 6 | 37 | M | III | colon carcinoma |
| 7 | 43 | F | IV | colon carcinoma |
| 8 | 55 | M | III | colon carcinoma |
| 9 | 50 | F | IV | colon carcinoma |
| 10 | 44 | F | II | rectal carcinoma |
| 11 | 43 | F | III | colon carcinoma |
| 12 | 69 | M | III | colon carcinoma |
| 13 | 47 | M | IV | colon carcinoma |
| 14 | 65 | F | III | colon carcinoma |
| 15 | 59 | M | II | rectal carcinoma |
| 16 | 47 | F | III | colon carcinoma |
