# Supplementary material for: miR-19a promotes colorectal cancer proliferation and migration by targeting TIA1
Source: Mol Cancer. 2017 Mar 4;16:53. doi: 10.1186/s12943-017-0625-8 (PMC5336638; doi:10.1186/s12943-017-0625-8)
Supplement: Additional file 3: Table S3. — Top 29 significantly upregulated miRNAs in the colon cancer tissues compared with the normal solid tissues analyzed by meta-analysis by using YM500. (DOCX 17 kb) [file 12943_2017_625_MOESM3_ESM.docx]

**miR-19a promotes colorectal cancer proliferation and migration by targeting TIA1**

**Supplemental table [3](http://www.ncbi.nlm.nih.gov/pmc/articles/PMC4505325/table/t1/). Top 29 significantly upregulated miRNAs in the colon cancer tissues compared with the normal solid tissues analyzed by meta-analysis by using YM500.**

| **rank** | **miRNA** | **fold change** |
| --- | --- | --- |
| 1 | hsa-miR-19a-3p | ∞ |
| 2 | hsa-miR-592 | ∞ |
| 3 | hsa-miR-628-5p | ∞ |
| 4 | hsa-miR-335-5p | ∞ |
| 5 | hsa-miR-7-5p | ∞ |
| 6 | hsa-miR-3613-5p | ∞ |
| 7 | hsa-miR-18a-5p | ∞ |
| 8 | hsa-miR-19b-3p | 356.6785756 |
| 9 | hsa-miR-32-5p | 256.5617914 |
| 10 | hsa-miR-135b-5p | 237.6108646 |
| 11 | hsa-miR-374a-3p | 192.8374551 |
| 12 | hsa-miR-142-5p | 176.6383137 |
| 13 | hsa-miR-590-5p | 176.484799 |
| 14 | hsa-miR-424-5p | 170.6609156 |
| 15 | hsa-miR-577 | 166.3526044 |
| 16 | hsa-miR-135a-5p | 147.7289561 |
| 17 | hsa-miR-142-3p | 134.5343833 |
| 18 | hsa-miR-21-5p | 98.97827767 |
| 19 | hsa-miR-542-3p | 92.86570816 |
| 20 | hsa-miR-33a-5p | 88.44081179 |
| 21 | hsa-miR-301a-3p | 80.37142035 |
| 22 | hsa-let-7f-2-3p | 75.17110578 |
| 23 | hsa-miR-153-5p | 74.01879661 |
| 24 | hsa-miR-374a-5p | 54.34889199 |
| 25 | hsa-miR-126-5p | 51.43912393 |
| 26 | hsa-miR-33b-5p | 51.00931989 |
| 27 | hsa-miR-203b-3p | 49.22311538 |
| 28 | hsa-miR-429 | 43.45666876 |
| 29 | hsa-let-7a-3p | 42.62088935 |
