# Supplementary figures and images for: miR-19a promotes colorectal cancer proliferation and migration by targeting TIA1
Source: Mol Cancer. 2017 Mar 4;16:53. doi: 10.1186/s12943-017-0625-8 (PMC5336638; doi:10.1186/s12943-017-0625-8)

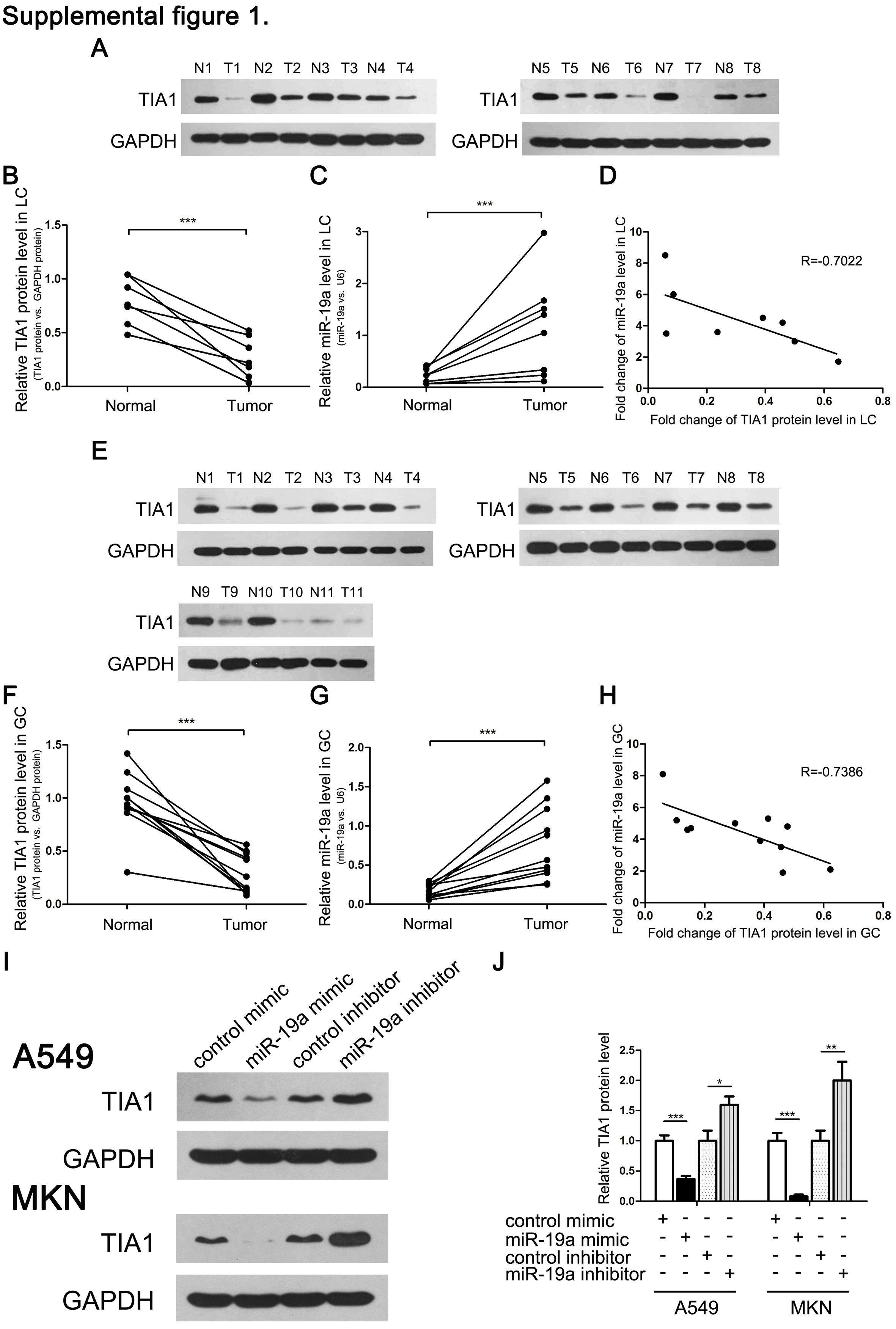

Supplement: Additional file 4: Figure S1. — miR-19a can also target TIA1 in LC and GC tissues and cell lines. (A and B) Western blot analysis of TIA1 protein levels in 8 paired LC (T) and normal adjacent tissue (N) samples. A: representative images; B: quantitative analysis. (C) Quantitative RT-PCR analysis of miR-19a levels in the same LC and normal adjacent tissue sample pairs. (D) Pearson’s correlation scatter plot of the fold changes of TIA1 protein and miR-19a levels in LC tissue pairs. (E and F) Western blot analysis of TIA1 protein levels in 11 paired GC (T) and normal adjacent tissue (N) samples. E: representative images; F: quantitative analysis. (G) Quantitative RT-PCR analysis of miR-19a levels in the same GC and normal adjacent tissue sample pairs. (H) Pearson’s correlation scatter plot of the fold changes of TIA1 protein and miR-19a levels in GC tissue pairs. (I and J) Western blot analysis of TIA1 protein levels in A549 and MKN cells transfected with control mimic, miR-19a mimic, control inhibitor or miR-19a inhibitor. E: representative images; F: quantitative analysis. *P < 0.05; **P < 0.01; ***P < 0.001. (TIF 1406 kb) [file 12943_2017_625_MOESM4_ESM.tif]

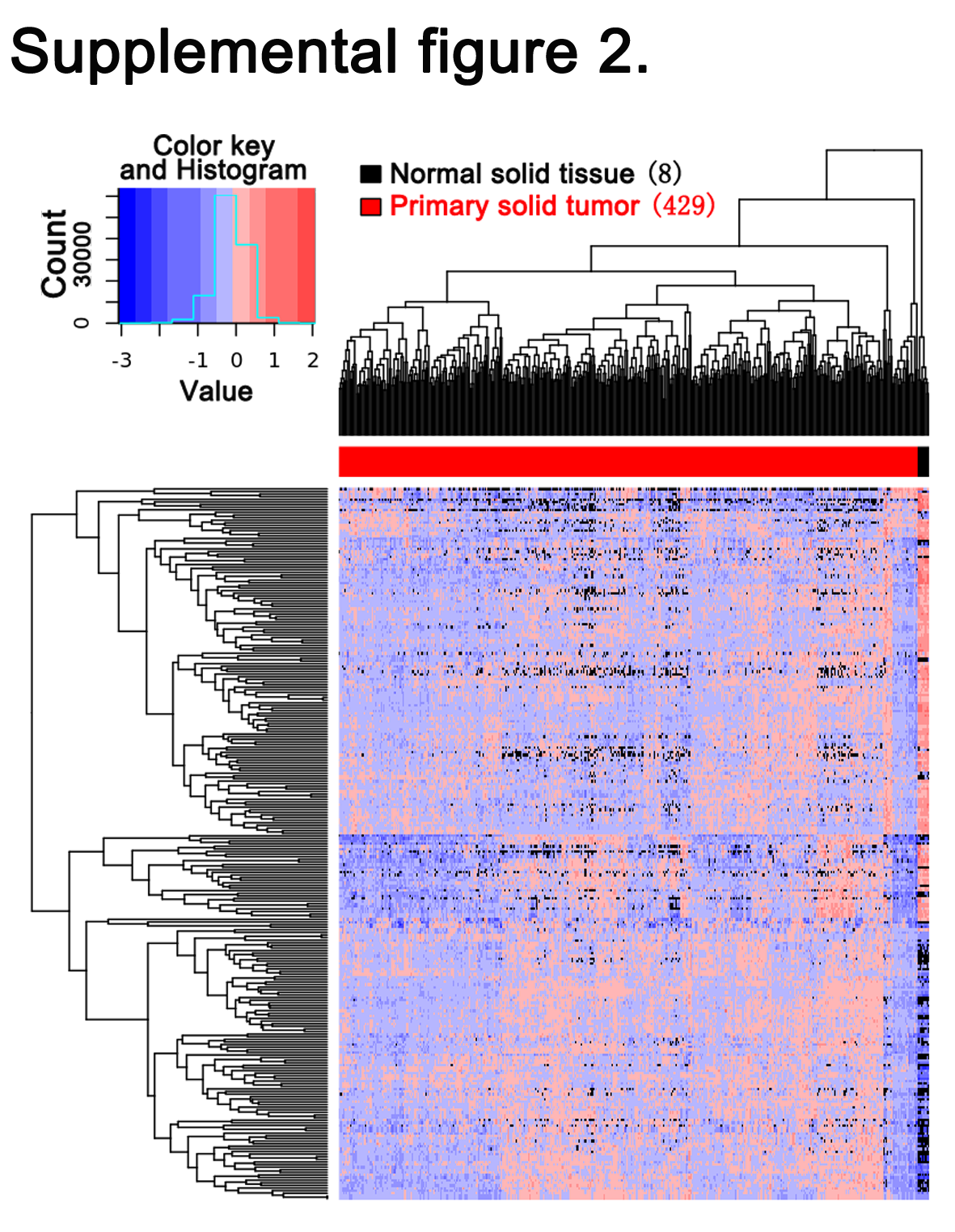

Supplement: Additional file 5: Figure S2. — Profiles of 273 significantly changed miRNAs in 429 primary solid colon tumors and 8 normal solid tissues from a meta-analysis by using YM500. Detailed information on these miRNAs is listed in Additional file 2: Table S2. (TIF 1762 kb) [file 12943_2017_625_MOESM5_ESM.tif]

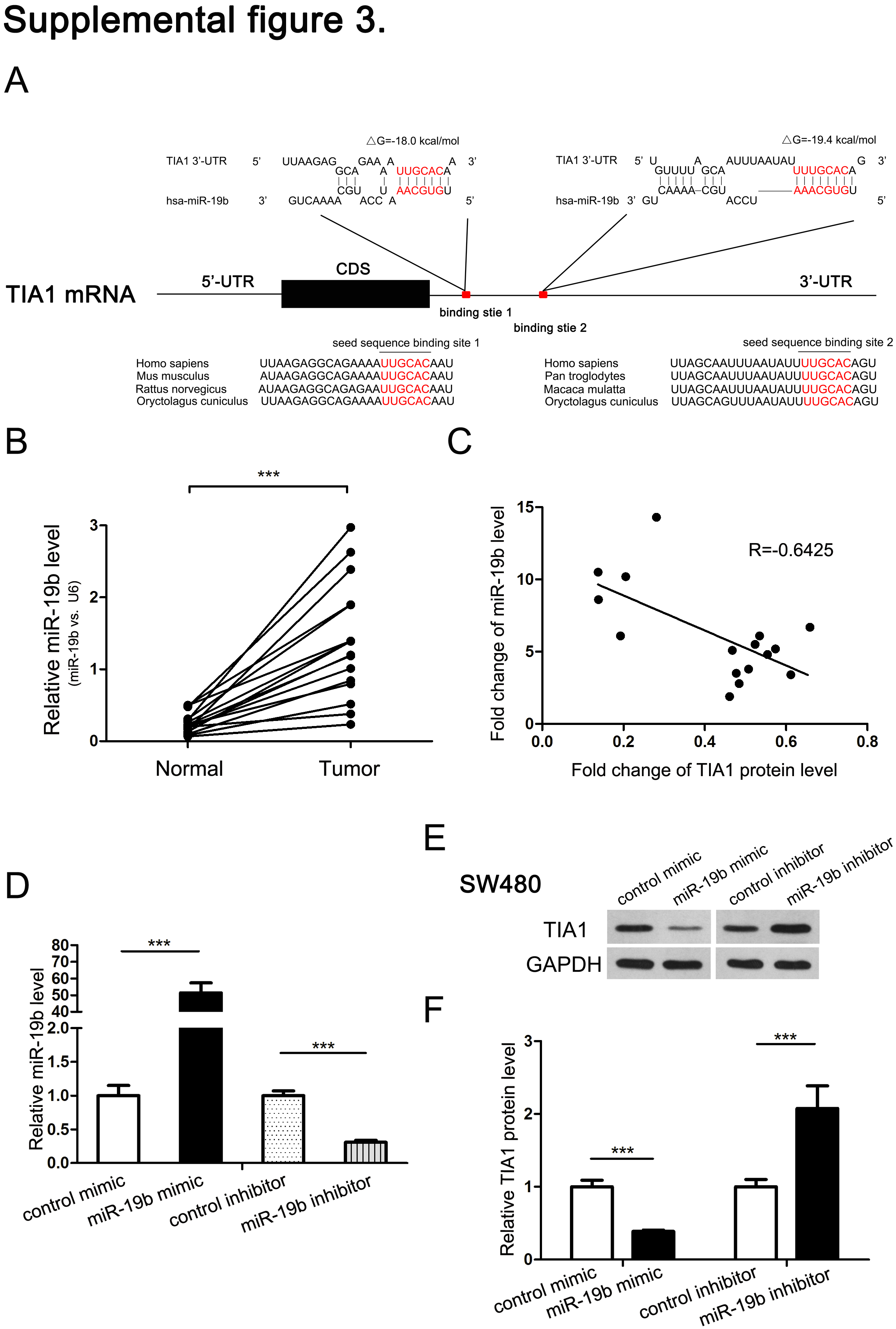

Supplement: Additional file 6: Figure S3. — miR-19b can also regulate TIA1 expression in CRC. (A) Schematic description of the hypothetical duplex formed by the interaction between the binding site in the TIA1 3’-UTR and miR-19b. The miR-19b seed sequence and the seed sequence binding sites in the TIA1 3’-UTR are indicated in red. All nucleotides of the seed sequence of the binding site are conserved in several species, including human, mouse, rat and rabbit. The predicted free energy values of the hybrids are indicated. (B) Quantitative RT-PCR analysis of miR-19b expression levels in the same 16 pairs of CRC and normal tissue samples. (C) Pearson’s correlation scatter plot of the fold change of miR-19b and TIA1 protein in human CRC tissue pairs. (D) Quantitative RT-PCR analysis of miR-19b levels in SW480 cells transfected with control mimic, miR-19b mimic, control inhibitor or miR-19b inhibitor. (E and F) Western blot analysis of TIA1 protein levels in SW480 cells transfected with control mimic, miR-19b mimic, control inhibitor or miR-19b inhibitor. E: representative images; F: quantitative analysis. ***P < 0.001. (TIF 917 kb) [file 12943_2017_625_MOESM6_ESM.tif]

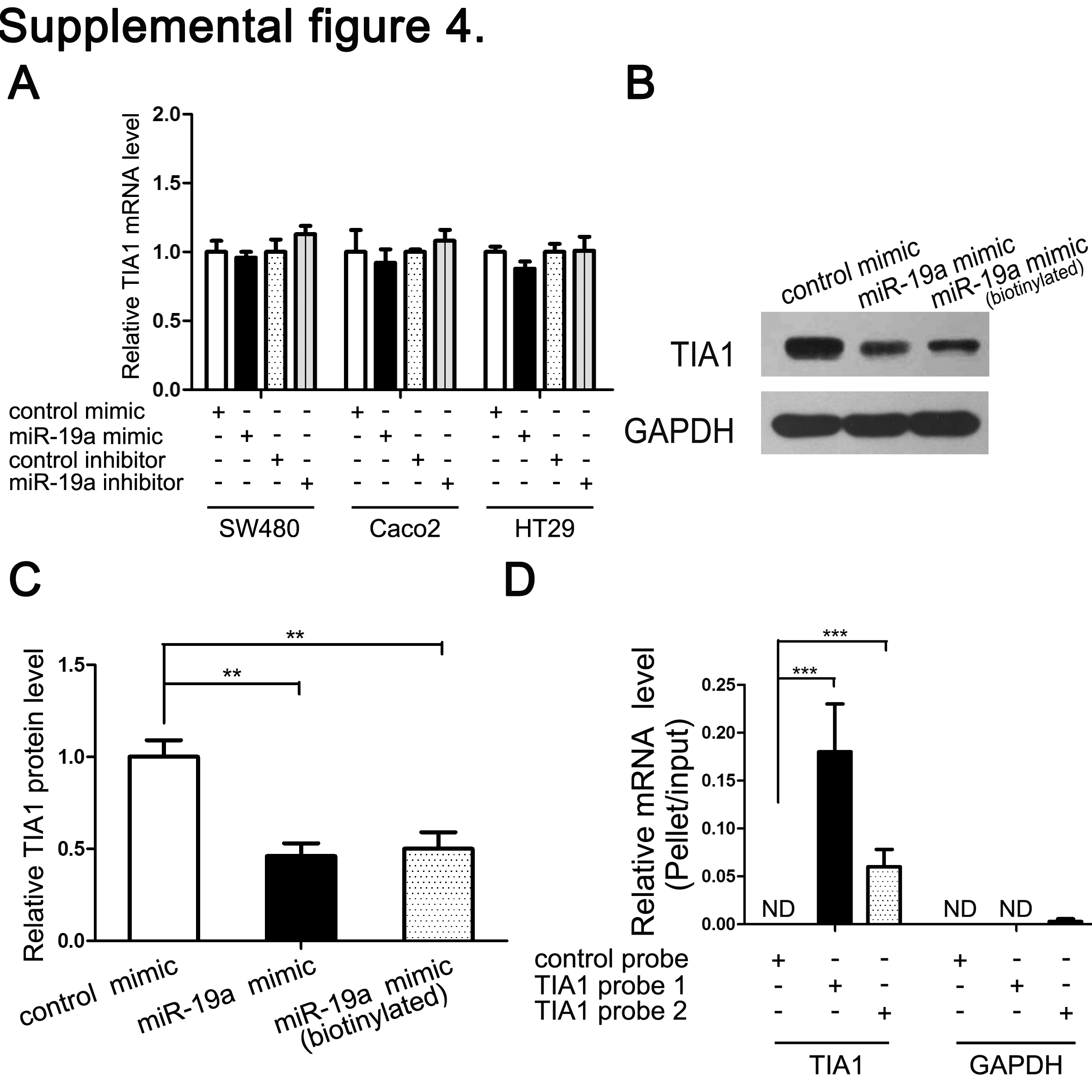

Supplement: Additional file 7: Figure S4. — Effect of miR-19a on TIA1 mRNA level and the efficacies of miR-19a probe and TIA1 mRNA probes. (A) Quantitative RT-PCR analysis of TIA1 mRNA levels in SW480, Caco2 and HT-29 cells transfected with control mimic, miR-19a mimic, control inhibitor or miR-19a inhibitor. (B and C) Western blot analysis of TIA1 protein levels in SW480 cells transfected with control mimic, miR-19a mimic or biotinylated miR-19a mimic. B: representative images; C: quantitative analysis. (D) Quantitative RT-PCR analysis of TIA1 and GAPDH mRNA levels in SW480 after pulling down with control probe or TIA1 mRNA probes. **P < 0.01; ***P < 0.001. (TIF 371 kb) [file 12943_2017_625_MOESM7_ESM.tif]

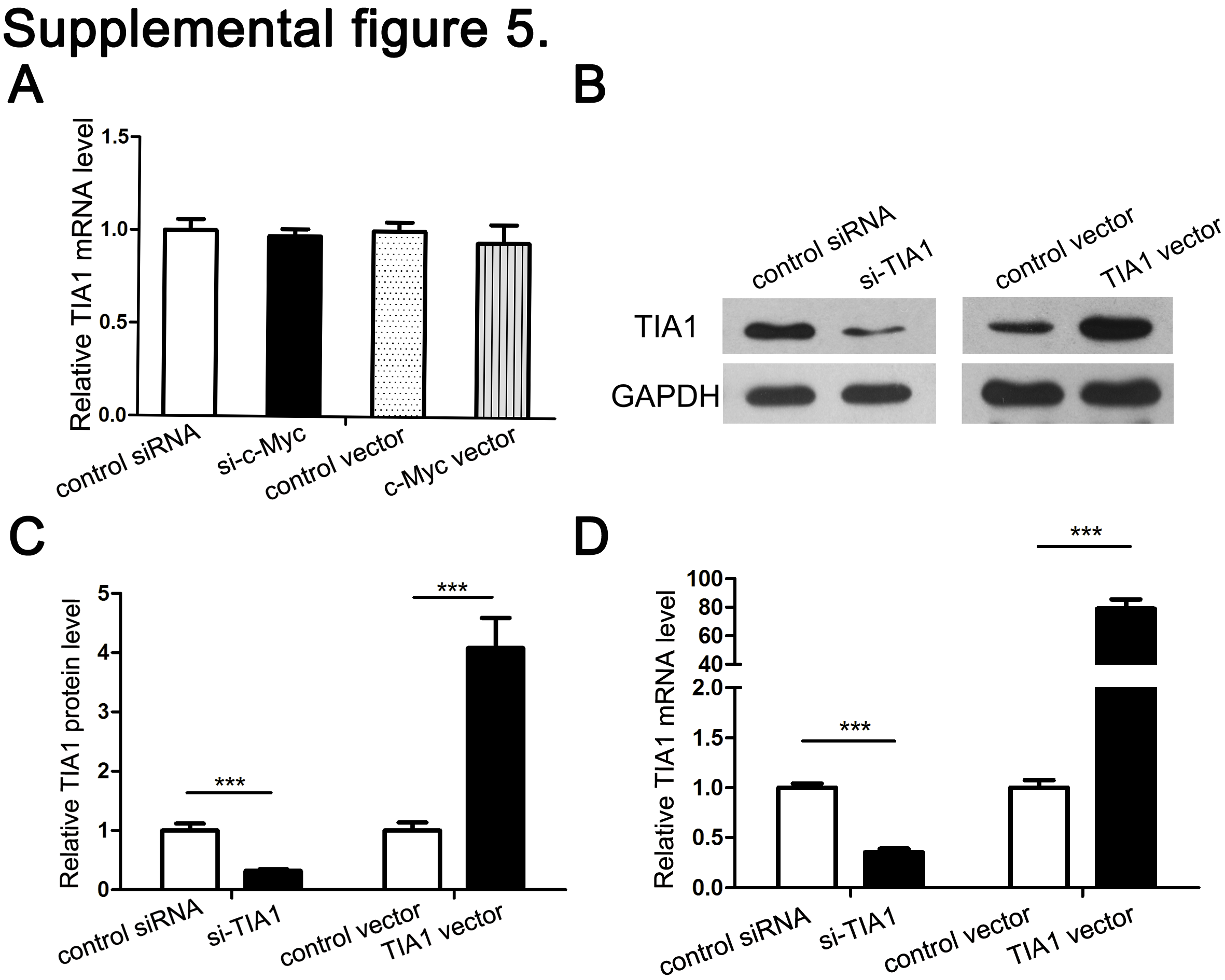

Supplement: Additional file 8: Figure S5. — Effect of c-Myc on TIA1 mRNA level and the efficacies of TIA1 siRNA and overexpression vector. (A) Quantitative RT-PCR analysis of TIA1 mRNA levels in SW480 cells transfected with control siRNA, si-c-Myc, control plasmid or c-Myc plasmid. (B and C) Western blot analysis of TIA1 protein levels in SW480 cells transfected with control siRNA, si-TIA1, control vector or TIA1 vector. B: representative images; C: quantitative analysis. (D) Quantitative RT-PCR analysis of TIA1 mRNA levels in SW480 cells transfected with control siRNA, si-TIA1, control vector or TIA1 vector. ***P < 0.001. (TIF 602 kb) [file 12943_2017_625_MOESM8_ESM.tif]

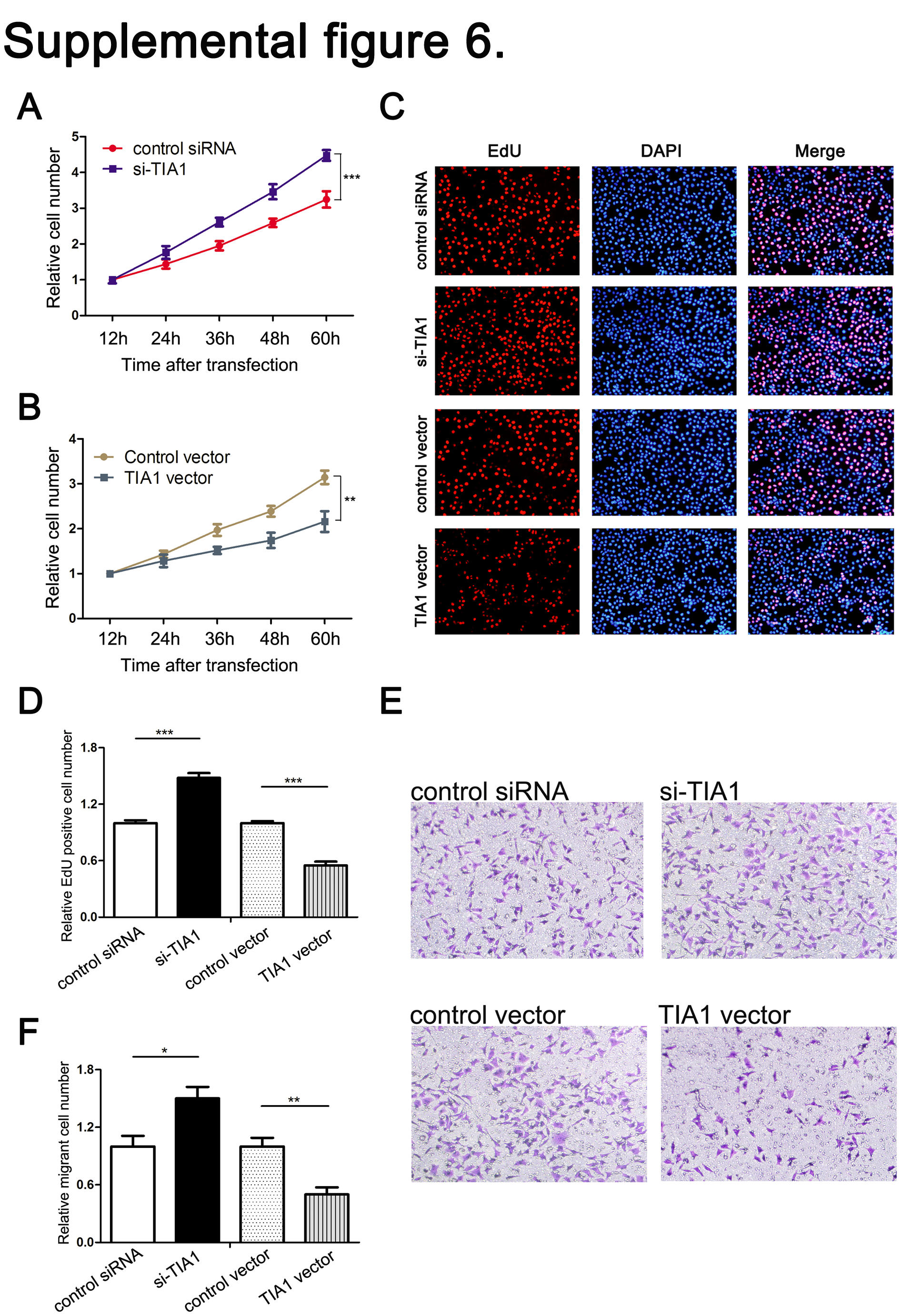

Supplement: Additional file 9: Figure S6. — Effects of TIA1 on SW480 proliferation and migration. (A and B) Cell proliferation assays (CCK-8) were performed 12, 24, 36, 48 and 60 h after the transfection of SW480 cells with equal doses of control siRNA, si-TIA1, control plasmid or TIA1 plasmid. (C and D) Cell proliferation assays (EdU) were performed in SW480 cells transfected with equal doses of control siRNA, si-TIA1, control plasmid or TIA1 plasmid. C: representative images; D: quantitative analysis. (E and F) Cell migration assays (transwell) were performed in SW480 cells that were transfected with equal doses of control siRNA, si-TIA1, control plasmid or TIA1 plasmid. E: representative images; F: quantitative analysis. *P < 0.05; **P < 0.01; ***P < 0.001. (TIF 4077 kb) [file 12943_2017_625_MOESM9_ESM.tif]

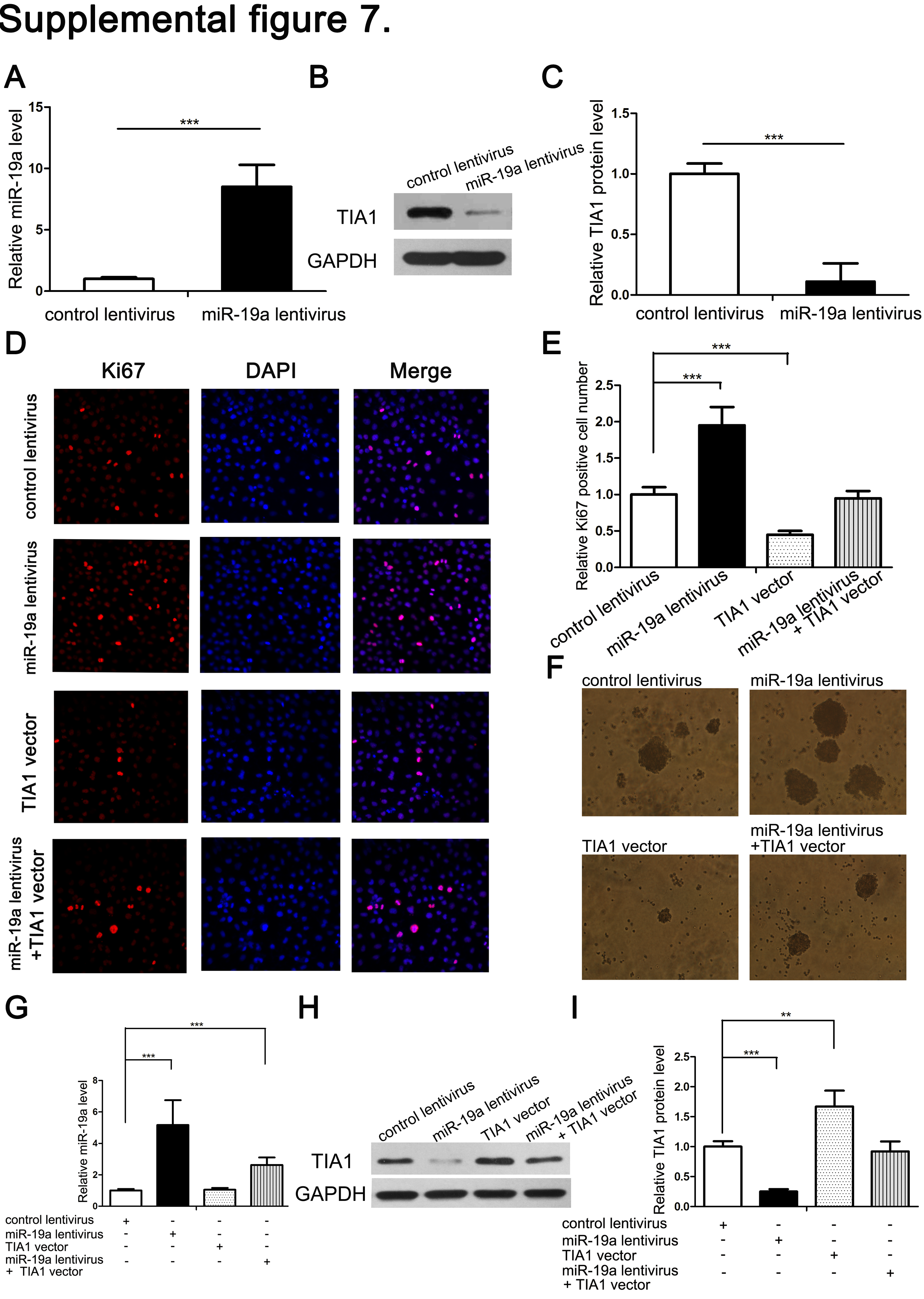

Supplement: Additional file 10: Figure S7. — The efficiency of miR-19a lentivirus and TIA1 vector on TIA1 protein level and SW480 proliferation. (A) Quantitative RT-PCR analysis of miR-19a levels in SW480 cells, which were infected with a control lentivirus or a lentivirus to overexpress miR-19a. (B and C) Western blot analysis of TIA1 protein levels in SW480 cells, which were infected with a control lentivirus or a lentivirus to overexpress miR-19a. B: representative images; C: quantitative analysis. (D-F) miR-19a could promote SW480 cell proliferation by targeting TIA1 in vitro. D representative images of Ki67 immunofluorescence; E: quantitative analysis of Ki67 immunofluorescence; F: representative images of formed SW480 colonies. (G) Quantitative RT-PCR analysis of miR-19a levels in tumors from implanted mice. (H and I) Western blot analysis of TIA1 protein levels in tumors from implanted mice. H: representative images; I: quantitative analysis. **P < 0.01; ***P < 0.001. (TIF 3290 kb) [file 12943_2017_625_MOESM10_ESM.tif]
